# Identification and validation of *SOCS1/2/3/4* as potential prognostic biomarkers and correlate with immune infiltration in glioblastoma

**DOI:** 10.1111/jcmm.17807

**Published:** 2023-06-14

**Authors:** Lirui Dai, Yongjie Han, Zhuo Yang, Yuling Zeng, Wulong Liang, Zimin Shi, Yiran Tao, Xianyin Liang, Wanqing Liu, Shaolong Zhou, Zhe Xing, Weihua Hu, Xinjun Wang

**Affiliations:** ^1^ Department of Neurosurgery The Fifth Affiliated Hospital of Zhengzhou University, Zhengzhou University Zhengzhou China; ^2^ Institute of Neuroscience, Zhengzhou University Zhengzhou China; ^3^ Henan International Joint Laboratory of Glioma Metabolism and Microenvironment Research Zhengzhou China; ^4^ Department of Blood Transfusion The Fifth Affiliated Hospital of Zhengzhou University Zhengzhou China

**Keywords:** biomarkers, gene expression profiling, glioblastoma, immune infiltration, *JAK/STAT3*, *SOCS1/2/3/4*

## Abstract

Suppressor of cytokine signalling (*SOCS*) 1/2/3/4 are involved in the occurrence and progression of multiple malignancies; however, their prognostic and developmental value in patients with glioblastoma (GBM) remains unclear. The present study used TCGA, ONCOMINE, SangerBox3.0, UALCAN, TIMER2.0, GENEMANIA, TISDB, The Human Protein Atlas (HPA) and other databases to analyse the expression profile, clinical value and prognosis of *SOCS1/2/3/4* in GBM, and to explore the potential development mechanism of action of *SOCS1/2/3/4* in GBM. The majority of analyses showed that *SOCS1/2/3/4* transcription and translation levels in GBM tissues were significantly higher than those in normal tissues. qRT‐PCR, western blotting (WB) and immunohistochemical staining were used to verify that *SOCS3* was expressed at higher *mRNA* and protein levels in GBM than in normal tissues or cells. High *SOCS1/2/3/4 mRNA* expression was associated with poor prognosis in patients with GBM, especially *SOCS3*. *SOCS1/2/3/4* were highly contraindicated, which had few mutations, and were not associated with clinical prognosis. Furthermore, *SOCS1/2/3/4* were associated with the infiltration of specific immune cell types. In addition, *SOCS3* may affect the prognosis of patients with GBM through JAK/STAT signalling pathway. Analysis of the GBM‐specific protein interaction (PPI) network showed that *SOCS1/2/3/4* were involved in multiple potential carcinogenic mechanisms of GBM. In addition, colony formation, Transwell, wound healing and western blotting assays revealed that inhibition of *SOCS3* decreased the proliferation, migration and invasion of GBM cells. In conclusion, the present study elucidated the expression profile and prognostic value of *SOCS1/2/3/4* in GBM, which may provide potential prognostic biomarkers and therapeutic targets for GBM, especially *SOCS3*.

## 1 INTRODUCTION

Glioblastoma (GBM) is the most common, aggressive primary malignant brain cancer type in humans worldwide.^1^ GBM, whose cause is unknown, is common among Caucasians and Asians, with the worst survival and highest morbidity among Caucasians.^2^ GBM can occur at any age, and the incidence increases steadily with age.^3^ Currently, the overall survival (OS) time of patients with GBM can be extended via radiotherapy, chemotherapy, surgical treatment and immunotherapy, but it is not possible to reduce its high recurrence rate after treatment. Previous clinical trials have shown that the 5‐year survival rate of GBM is 4%–5%, and the 2‐year survival rate is 26%–33%.^4^ Hence, it is necessary to further explore the carcinogenic mechanism of GBM and provide better prognostic assessment strategies.

Tumour RNA sequencing (RNA‐seq) data are increasingly being used to identify gene signatures and clinical prognostic factors, including gender, age, GBM tumour grade and Karnofsky score.^5^ Although previous studies have explored the prognostic factors of glioma, the prognosis of patients with glioma remains poor. Current high‐throughput sequencing technology provides rapid analysis of biomarkers and in‐depth exploration of potential mechanisms of disease.^6^ It is important to find biomarkers that affect the survival and prognosis of glioma patients as soon as possible. The present study explored the influence of four genes in the suppressor of cytokine signalling (*SOCS*) family on GBM and experimentally evaluated on the expression and functional influence of *SOCS3* in the *SOCS* family in GBM.

The *SOCS* family consists of eight members, whose dominant role is to inhibit cytokine signal transduction.^7^ *SOCS1* and *SOCS3* can inhibit the signal transduction of various cytokines such as interleukin‐6 (IL6), leukaemia inhibitor factor (LIF), OSM, INF gamma and growth hormone (GH), and play regulatory roles in the activation of various immune responses and the pathogenesis of tumours in vivo.^8^ *SOCS2* is induced by a variety of cytokines that activate *STAT5*, including GH, IL‐6 and LIF, and it is involved in the ubiquitination of target proteins, including GHR and a variety of signalling proteins.^9^ *SOCS4* is an important regulator of antiviral immunity.^10^ As one of the negative feedback loops of the JAK/STAT signalling pathway, *SOCS4* can decrease the *STAT3* signalling of EGFR by increasing receptor degradation.^11^ *SOCS5*, *SOCS6*, *SOCS7* and *CIS* play protumour or antitumour roles in a variety of cancers, and may affect the progression of tumours via different mechanisms. For example, *SOCS5* can inhibit the migration and invasion of hepatocellular carcinoma cells in vitro by activating PI3K/Akt/mTOR‐mediated autophagy.^12^ It is worth mentioning that the expression alterations of *SOCS1/2/3/4* negatively regulate the JAK/STAT signalling pathway in many tumour cells, so *SOCS1/2/3/4* is the main research content of this article. In the current study, *SOCS1/2/3/4* were regarded as the key genes affecting the survival and prognosis of patients with GBM, while *SOCS3*, as a negative regulator, could regulate the *JAK/STAT3* signalling pathway and inhibit tumour cell proliferation and tumour development.^13^ Therefore, *SOCS3* was included in the current study for further experimental verification.

## 2 MATERIALS AND METHODS

### 2.1 Oncomine analysis

Oncomine (https://www.oncomine.org/) gene expression microarray database was used to analyse genome‐wide expression relevant research.^14^, ^15^ Oncomine was employed to explore *SOCS1/2/3/4* RNA levels in brain and central nervous system (CNS) cancer. After retrieving and analysing the Oncomine database, correlation analysis results on *SOCS1/2/3/4* genes were included. Among them, *SOCS1/2/3/4* genes with significant differences were selected for further analysis. The screening criteria were as follows: (1) gene: *SOCS1/2/3/4*; (2) cancer type: Brain and CNS Cancer; (3) data type: all; (4) analysis type and target: brain and CNS cancer vs. normal tissue; (5) *p* < 0.0001, fold‐change>2, gene rank = top 10%. Significant difference was set at *p* < 0.05.

### 2.2 Timer2.0 analysis

‘Immune module’ of the TIMER2.0 (http://timer.cistrome.org/) database^16^ allows us to analyse immune infiltration estimations for *SOCS1/2/3/4* expression by TIMER and CIBERSORT algorithms. Six immune cell types were performed using TIMER algorithms, including lymphocytes, macrophages, NK cells and neutrophils, etc.

### 2.3 Analysis of UALCAN

TCGA gene expression data were analysed by the University of ALabama at Birmingham CANcer (UALCAN) (http://ualcan.path.uab.edu/)^17^ database to observe *SOCS1/2/3/4* and their hub genes' expression in GBM. In addition, the association between *SOCS1/2/3/4* and GBM clinical and pathological features (such as race, IDH status or OS, etc.) and their potential prognostic significance were observed.

### 2.4 SangerBox analysis

SangerBox3.0 (http://sangerbox.com/home.html)^18^ was used for prognostic analysis of gene expression, immune cell analysis, immunomodulatory gene analysis, immune checkpoint gene analysis and mutational landscape analysis. Data source: TCGA and GTEx databases. Data transformation: log_2_(x + 0.001). Survival data included overall survival (OS), disease‐specific survival (DSS) and progression‐free interval (PFI). Correlation coefficient: Pearson.

### 2.5 Analysis of biological function

Gene expression data of GBM in HTSeq‐FPKM were acquired from TCGA data set to explore 543 patients with GBM. Pearson's correlation coefficient (|*r*| > 0.4 and *p* < 0.001) to screen the coexpressed genes of *SOCS1/2/3/4*. Moreover, Gene Ontology (GO) and Kyoto Encyclopaedia of Genes and Genome (KEGG) analysis^19^ of coexpressed genes were evaluated by employing the R ‘cluster profiler’ software package to search the possible biological functions of *SOCS1/2/3/4*. Coexpressed genes of the *SOCS1/2/3/4* protein–protein interaction (PPI) network was built by employing the GeneMANIA (http://genemania.org/) database.^20^ The PPI network was put into Cytoscape 3.6.1,^21^ and the cytoHubba plug‐in was used as the screening criteria for hub genes. In total, 10 genes with the highest correlation were considered to be hub genes, and relevant analysis was subsequently conducted.

### 2.6 Human Protein Atlas (HPA) analysis

The subcellular localization of *SOCS1/2/3/4*, as well as their expression in GBM were analysed by immunohistochemistry employing the HPA database (https://www.proteinatlas.org/).^22^

### 2.7 Linked Omics database analysis

Linked Omics Database (http://www.linkedomics.org/login.php)^23^ contains multiomics data and clinical data for 32 cancer types, as well as data for a total of 11,158 patients from the TCGA project. It is also a multiomics database that integrates proteomics data from mass spectrometry (MS) for selected TCGA tumour sample. The differentially expressed genes (DEGs) related to *SOCS3* were screened from TCGA‐GBM cohort by the LinkFinder module, and the Pearson correlation coefficient was employed to obtain the results. The results were shown as volcano plots and heat maps.

### 2.8 TISDB analysis

The association between the immune system and GBM was explored via the TISIDB database (http://cis.hku.hk/TISIDB/),^24^ and the correlation between *SOCS1/2/3/4* expression in GBM and immune infiltration was evaluated by Spearman's correlation analysis.

### 2.9 Genetic alterations of SOCS1/2/3/4 in GBM

The cBioPortal database (https://www.cbioportal.org/)^25^, ^26^ was employed to explore the genetic alterations, gene alteration frequencies and copy number alterations of *SOCS1/2/3/4* in GBM.

### 2.10 Clinical tissue specimens

The tissues of 23 patients with low‐grade glioma (LGG) and GBM (including 12 patients with LGG and 11 patients with GBM) used for reverse transcription‐quantitative PCR (RT‐qPCR) and immunohistochemical staining were obtained from the Fifth Affiliated Hospital of Zhengzhou University (Zhengzhou, China). The studies involving patients were reviewed and approved by the Ethics Review Committee of the Fifth Affiliated Hospital of Zhengzhou University (approval no. KY2021004).

### 2.11 Cell cultures and transfections

The human GBM cell lines U87‐MG, T89G, U251, U118, LN229, A172 and LN18 were cultured in DMEM medium with 10% foetal bovine serum (FBS) (Biological Industries; Sartorius AG) at 37°C in the presence of 5% CO_2_. The small interfering RNAs (siRNAs), including three different *SOCS3* siRNA sequences and a control siRNA, were purchased from Guangzhou RiboBio Co., Ltd., and were transfected into cells with Lipofectamine® 3000. *SOCS3* plasmid was purchased from Guangzhou Dahong Biotechnology Co., LTD.

### 2.12 RT‐qPCR assay

Total RNA was extracted from patients' tissue employing TRIzol® (Takara Bio, Inc.) and other reagents such as chloroform, isopropyl alcohol and 75% ethanol. PrimeScript™ RT reagent Kit with gDNA Eraser and TB Green Premix Ex Taq™ II (Takara Bio, Inc.) were used to reversely transcribed RNA into cDNA and for RT‐qPCR. The primers used were as follows: *SOCS3*, forward, 5′‐CACCTGGACTCCTATGAGAAAGTCA‐3′, reverse, 5′‐ GGGGCATCGTACTGGTCCAGGAA ‐3′, GAPDH, forward, 5′‐ CAGGAGGCATTGCTGATGAT −3′, reverse, 5′‐ GAAGGCTGGGGCTCATTT −3′.

### 2.13 Western blotting

Cells were washed in PBS, detached with a cell scraper and centrifuged for 10 min at 12,000 × g at 4°C. Cell lysates were boiled for 15 min at 100°C. Total protein (15–20 μg) was electrophoresed by SDS‐PAGE (cat. no. P0012; Epizyme Biotech) and transferred to PVDF membranes (Millipore Sigma) for 100 min, followed by overnight incubation at 4°C with primary antibody against *SOCS3* (rabbit monoclonal antibody [mAb]; cat. no. #52113; 1:1000; Cell Signaling Technology, Inc.), (cat. no. #AB16030; 1:100; Abcam), proliferating cell nuclear antigen (PCNA; rabbit mAb; cat. no. #13110; 1:1000; Cell Signaling Technology, Inc.), β‐actin (cat. no. #66009‐1‐lg; 1:5000; Protein Tech Group, Inc.), β‐tubulin (cat. no. #M20005M; 1:5000; Abmart Pharmaceutical Technology Co., Ltd.). The PVDF membrane was then washed with TBST‐Tween 20 (cat. no. #T1087; Beijing Solarbio Science & Technology Co., Ltd.) three times for 15, 10 and 5 min, respectively, and incubated for 2 h at room temperature with a goat antirabbit IgG secondary antibody (Affinity HRP; 1:5000). Finally, the PVDF membrane was washed for 15, 10 and 5 min, and then visualized with enhanced chemiluminescence using the Superstar ECL Plus Ready‐to‐use (cat. no. AR1171, lot no. 16H31C71; Boster Biological Technology).

### 2.14 Colony formation assay

Transfected cells were digested with trypsin and counted with cell technology plates. Next, 2 mL medium containing 10% FBS was placed into a 6‐well plate, and 500 cells were added to the 6‐well plate. The cells were collected after 10 days of culture, fixed in paraformaldehyde for 30 min, stained with crystal violet for 30 min, washed with PBS, observed and counted.

### 2.15 Transwell assay

The Transwell chamber and the Matrigel were purchased from Corning Inc and Biozellen, respectively. For migration experiments, the upper layer of each chamber was inoculated with 30,000 cells, and 100 μL serum‐free medium and 800 μL 10% FBS medium were added to the lower layer of the chamber. For the invasion assay, Matrigel was added to the upper layer of the chamber, and the subsequent steps were performed as aforementioned.

### 2.16 Wound healing assay

A total of 15, 000 cells were planted into 6‐well plates. When transfected cells reached 80% confluence, scratches were produced in the cell monolayer in the middle of each well using a 200 μl pipette tip. The cells were washed using PBS and placed under an inverted microscope (magnification, ×40; Nikon Corporation) for observation and photography. Next, 2 mL medium containing 10% FBS was added to each well. After 48 h, the 6‐well plate was removed, washed with PBS and photographed under an inverted microscope. The migration distance was calculated as follows: Migration distance = Scratch width observed at 0 h—scratch width observed at 48 h.

### 2.17 Immunohistochemical staining analysis

Tissue samples of 12 patients with LGG and 11 patients with GBM were fixed with formaldehyde, paraffin embedded, sectioned and incubated with anti‐*SOCS3* antibodies overnight at 4°C, followed by incubation with a sheep antirabbit secondary antibody for 20 min at room temperature. Sample was then washed with PBS and incubated with a streptomycin working solution at room temperature for 15 min. Next, DAB chromogenic solution was used, and areas with a strong immune response were selected and observed at ×400 magnification.

### 2.18 Statistical analysis

GraphPad Prism 9 (GraphPad Software, Inc.) was employed for creating graphs, while SPSS 26.0 (IBM Corp) was employed for statistical analysis. Normally distributed data were expressed as the mean ± standard deviation. Student's *t*‐test was employed to compare *SOCS1/2/3/4* expression in cancer and normal tissues, as well as the proliferation, invasion and migration of GBM cells before and after knocking down *SOCS3*. Kaplan–Meier curves were employed to analyse patients' survival according to their *SOCS1/2/3/4* expression levels. Univariate Cox regression analysis was employed to calculate hazard ratio (HR) and 95% confidence interval (CI) in survival analysis. All R packages used were R software version V4.0.3.

## 3 RESULTS

### 3.1 *SOCS1/2/3/4* expression is increased in GBM

**FIGURE 1:**
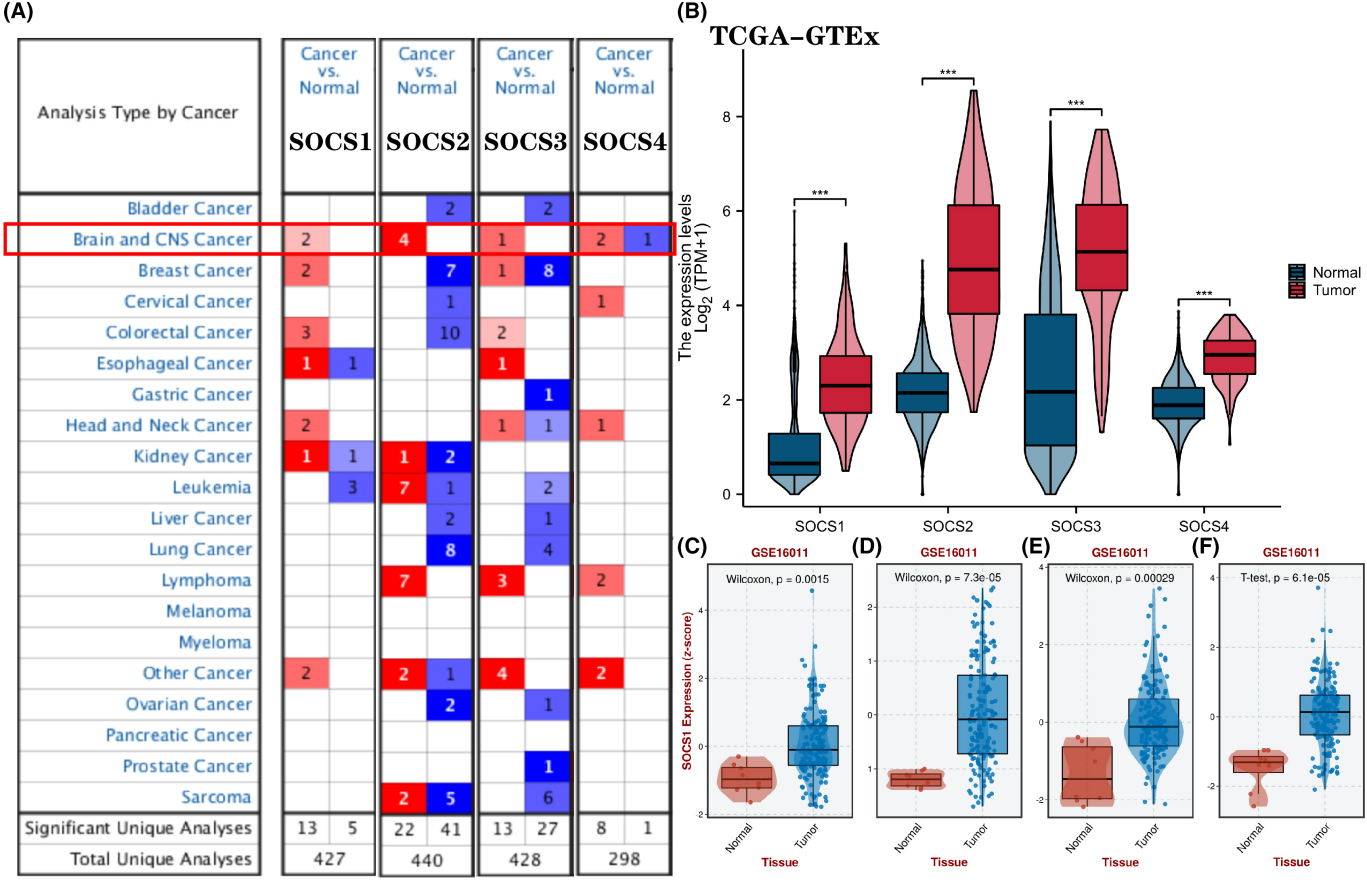
(A). *SOCS1/2/3/4 mRNA* expression levels in the Oncomine database in tumours. Red represents overexpressed genes, while blue represents underexpressed genes. The threshold parameters were as follows: *p* = 1 × 10^−4^ and fold‐change =1.5. (B). TCGA‐GTEx databases showed that *SOCS1/2/3/4* expression in normal and GBM tissues. (C–F). GSE16011 data set was used to analyse *SOCS1/2/3/4* expression in normal and tumour tissues. The horizontal line in the middle of each box plot represents the median. When the median in the red box plot is higher than the median in the blue box plot, it means that the gene expression level in the cancer tissue is higher than that in the normal tissue, otherwise it is the opposite. (*p < 0.05; **p < 0.01; ***p < 0.001). *SOCS*, suppressor of cytokine signalling.

**FIGURE 2:**
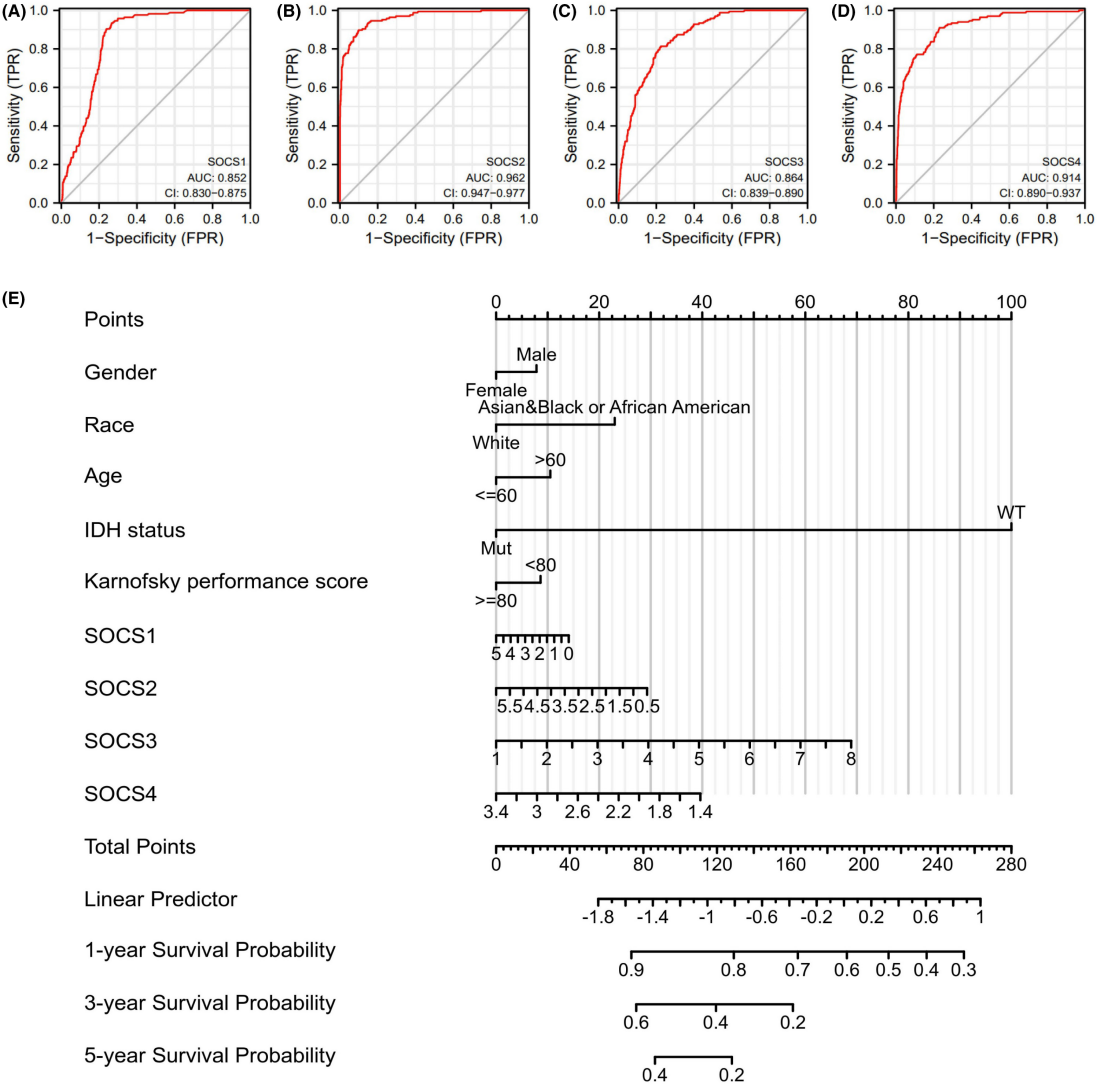
The ROC curve to test the value of *SOCS1/2/3/4* to identify GBM tissues was constructed. (A) *SOCS1*; (B) *SOCS2*; (C) *SOCS3*; (D) *SOCS4*. The results were 0.852, 0.962, 0.864 and 0.914, respectively, which suggests that *SOCS1/2/3/4* genes may have the potential to identify GBM samples. (E) Nomogram model predicting the 1‐, 3‐ and 5‐year overall survival in patients with GBM. The nomogram is employed by summing all points identified on the scale for each variable, including gender, race, age, IDH status, Karnofsky performance score and *SOCS1/2/3/4* expression in GBM. The total points projected on the bottom scales reveal the probabilities of 1‐, 3‐and 5‐year overall survival.

The Oncomine database was employed to compare *SOCS1/2/3/4 mRNA* expression between brain and CNS cancer and normal tissues. The results revealed that *SOCS1*, *SOCS2*, *SOCS3* and SOCS4 *mRNA* levels were upregulated in patients with brain and CNS cancer (Figure 1A). In‐depth analysis revealed that *SOCS1* was overexpressed in patients with GBM, with a fold‐change of 2.133 and a *p* = 5.37 × 10^−9^. In the Sun database, the *SOCS1* expression level was high in GBM with a fold‐change of 2.025 and a *p* = 8.83 × 10^−12^. In the Murat data set, *SOCS2 mRNA* expression was increased in patients with GBM, with a fold‐change of 3.344, and *p* = 1.36 × 10^−18^. In the TCGA and Lee databases, the *mRNA* levels of *SOCS2* were upregulated in GBM, with a fold‐change of 3.164 and 3.139, and *p*‐values of 2.01 × 10^−14^ and 5.22 × 10^−5^, respectively. In the Berdel database, the *mRNA* levels of *SOCS3* were increased in GBM, with a fold‐change of 5.701 and *p* = 3.96 × 10^−7^. Moreover, *SOCS4* was upregulated in anaplastic astrocytoma and oligodendroglioma in the Sun database, with fold‐changes of 3.062 and 2.357, and *p*‐values of 4.01 × 10^−7^ and 1.61 × 10^−5^ (Table 1). In addition, TCGA‐GTEx database and GSE16011 data set in GEO database^27^ were used to observe *SOCS1/2/3/4* expression differences between GBM and normal tissues. All the results showed that mRNA levels of *SOCS1/2/3/4* were significantly higher in GBM than in normal tissues (Figure 1B–F **)**. The receiver operating characteristic curves were employed to analyse the efficacy of *SOCS1/2/3/4* to distinguish between GBM and normal brain samples. The results were 0.852, 0.962, 0.864 and 0.914, respectively, these suggests that *SOCS1/2/3/4* genes may possess the potential to identify GBM samples (Figure 2A–D). In addition, a nomogram model that contained multiples important predictors in the Cox analysis was conducted to predict the 1‐, 3‐ and 5‐ year survival rate of patients with GBM. For example, a male patient (8 points) from Asia (0 points), >60 years old (10 points), IDH status: WT (100 points); Karnofsky performance score:<80 (8 points), *SOCS1*: 5 (0 points), *SOCS2*: 5 (0 points), *SOCS3*: 2 (10 points) and *SOCS4*: 3.4 (0 points). Thus, the total score is 136 points; the patient's 1‐year survival rate is about 78%, the 3‐year survival rate is about 32% and the patient's 5‐year survival rate is 0. The results also revealed that IDH status and *SOCS3* contributed the most to the total points and survival probability relative to the other factors in the multivariate regression model (Figure 2E).

**TABLE 1:** Expression of *SOCS1/2/3/4* in glioma in the Oncomine database.

Name	Types of Glioma vs. Normal	Fold‐change	*t*‐test	*p* value	Reference
SOCS1	Glioblastoma vs. Normal	2.133	12.532	5.37E−9	TCGA
SOCS1	Glioblastoma vs. Normal	2.025	8.210	8.83E−12	Sun
SOCS2	Glioblastoma vs. Normal	3.344	11.908	1.36E−18	Mura
SOCS2	Glioblastoma vs. Normal	3.164	15.962	2.01E−14	TCGA
SOCS2	Glioblastoma vs. Normal	3.139	4.714	5.22E−5	Lee
*SOCS3*	Glioblastoma vs. Normal	5.701	8.703	3.96E−7	Bredel
SOCS4	Anaplastic Astrocytoma vs. Normal	3.062	5.864	4.01E−7	Sun
SOCS4	Oligodendroglioma vs. Normal	2.357	4.628	1.61E−5	Sun

Abbreviations: GBM, glioblastoma; SOCS, suppressor of cytokine signalling.

### 3.2 High *SOCS1/2/3/4* expression is closely associated with WHO status, histological types, 1p/19q codeletion, primary therapeutic effects and prognosis of patients with GBM

**FIGURE 3:**
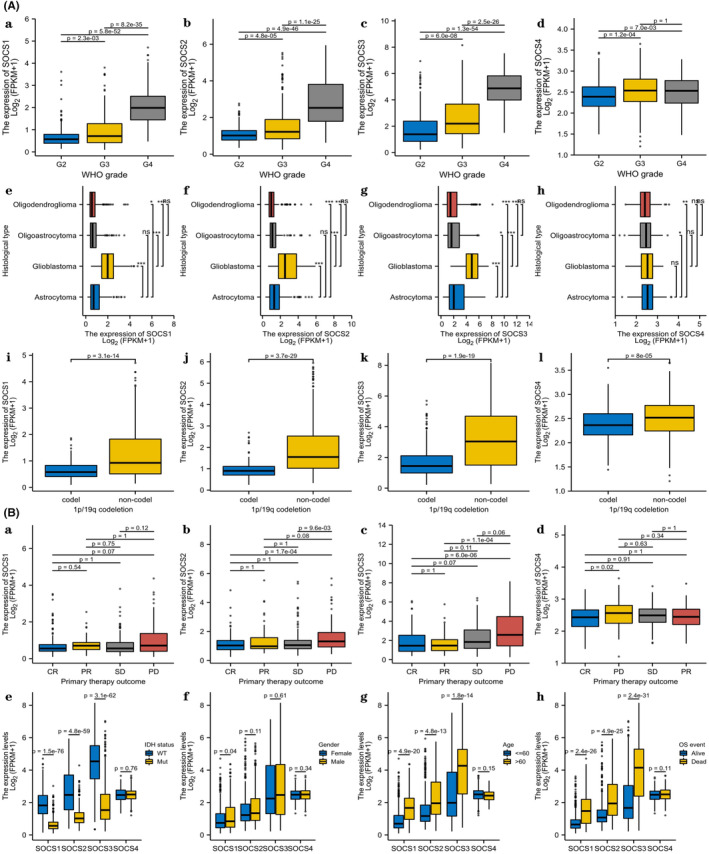
Expression and clinicopathological parameters of *SOCS1/2/3/4* in patients with glioma in the UALCAN database. A (a–d) Association between expression of *SOCS1/2/3/4* and WHO grade; A (e–h) Association between expression of *SOCS1/2/3/4* and histological grade; A (i–l) Association between expression of *SOCS1/2/3/4* and 1p/19q co‐deletion; B (a–d) Association between expression of *SOCS1/2/3/4* and primary therapy outcome; B (e–h) Association between expression of *SOCS1/2/3/4* and IDH status, gender, age and OS, respectively. G2, G3: low‐grade glioma; G4: GBM, glioblastoma. CR, complete response; PR, partial response; SD, stable disease; PD, progressive disease.

**FIGURE 4:**
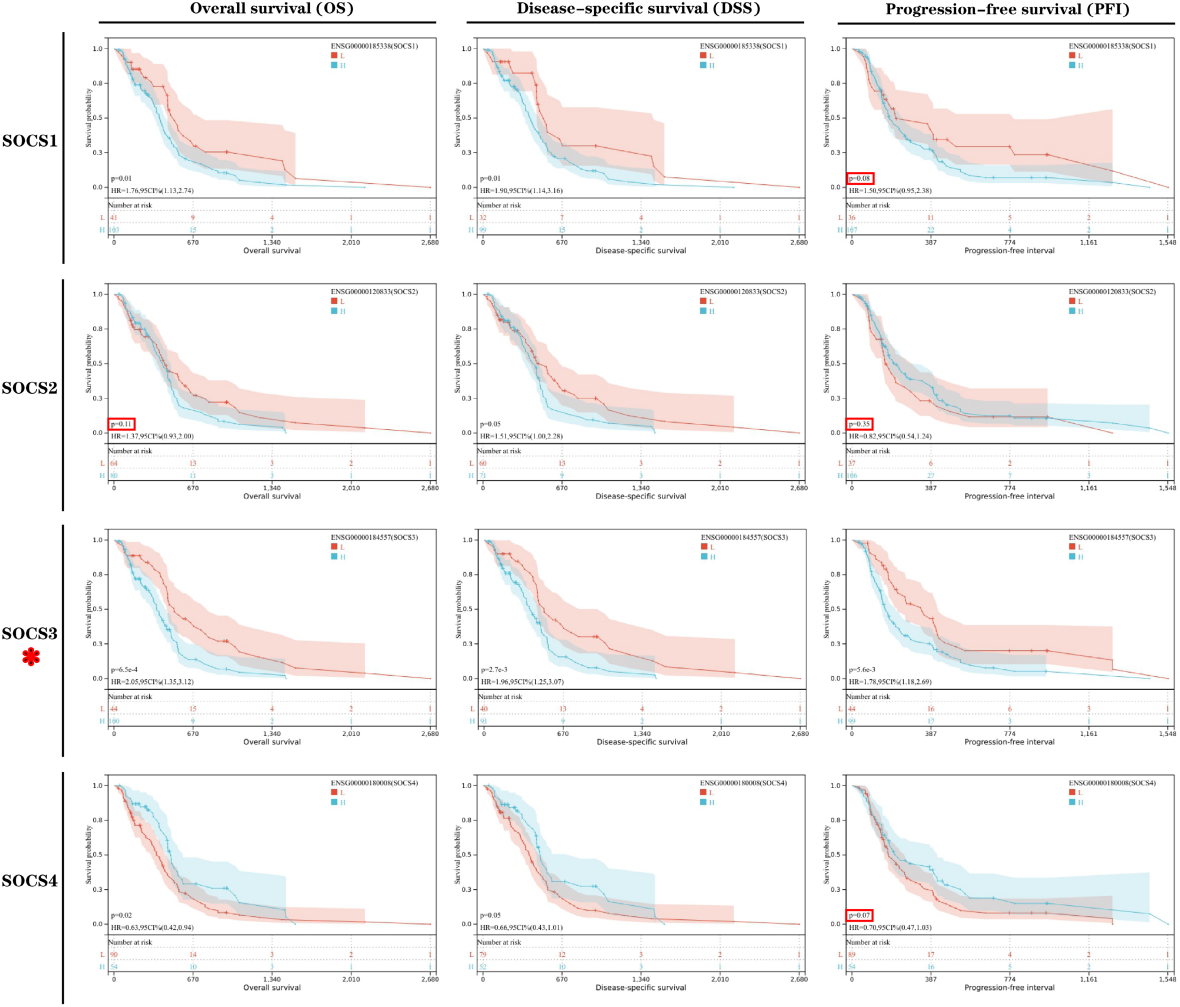
Survival curve analysis of the prognostic significance of high and low SOCS1/2/3/4 expression in glioma using Sangerbox3.0 database. Overall survival: OS, disease‐specific survival: DSS and progression‐free interval: PFI.

Analysis of the UALCAN database revealed that high *SOCS1/2/3/4* expression was associated with the clinicopathological characteristics and prognosis of patients with glioma. High *SOCS1* expression was associated with World Health Organization (WHO) status, histological type, IDH status, 1p/19q co‐deletion, age, gender and overall survival (OS). High *SOCS2* expression was significantly associated with WHO status, histological type, IDH status, 1p/19q codeletion, age and OS. High *SOCS3* expression was positively correlated with WHO status, histological type, IDH status, 1p/19q codeletion, primary therapeutic effects and age and negatively correlated with OS. High *SOCS4* expression was associated with WHO status, 1p/19q co‐deletion and primary therapeutic effects (*p* < 0.05; Table 2) (Figure 3A,B). Furthermore, we analysed the prognostic value of *SOCS1/2/3/4* expression in GBM using sangerbox3.0 database, the results revealed that high *SOCS3* expression possessed a poorer patient OS, DSS and PFI than that of low *SOCS3* expression (*p* < 0.05; Figure 4), whereas high expression of SOCS1 and SOCS4 in GBM was associated with poor OS and DSS, and high expression of SOCS2 was only associated with poor DSS (Figure 4). These results suggested that SOCS1/2/3/4 were closely associated with clinicopathological features and may be the oncogenes in GBM. In particular, SOCS3 has the greatest prognostic value for GBM patients, which also prepares us for further experimental research on SOCS3.

**TABLE 2:** Association between *SOCS1/2/3/4* expression and clinicopathological characteristics of patients with glioma in the UALCAN database. *SOCS1/2/3/4*, suppressor of cytokine signalling 1/2/3/4; G2, G3: low‐grade glioma; G4: GBM, glioblastoma.

Clinicopathologic features		SOCS1 (*p* value)	SOCS2 (*p* value)	*SOCS3* (*p* value)	SOCS4 (*p* value)
WHO status	G2 vs. G3	2.30E−3	4.89E−5	6.05E−8	1.23E−4
	G2 vs. G4	5.86E−52	4.91E−46	1.39E−54	7.03E−3
	G3 vs. G4	8.26E−35	1.15E−25	2.51E−26	1.00
Histological type	Astrocytoma vs. Glioblastoma	5.38E−4	3.53E−5	2.46E−7	0.703
	Astrocytoma vs. Oligoastrocytoma	0.476	0.156	4.52E−2	3.70E−2
	Astrocytoma vs. Oligodendroglioma	1.52E−2	3.68E−7	1.47E−3	5.14E−3
	Glioblastoma vs. Oligoastrocytoma	4.26E−5	4.67E−9	5.38E−11	0.365
	Glioblastoma vs. Oligodendroglioma	2.86E−4	2.48E−12	2.31E−7	0.147
	Oligoastrocytoma vs. Oligodendroglioma	0.583	0.442	0.810	0.990
IDH	WT vs. Mut	1.53E−76	4.81E−59	3.11E−62	0.759
1p/19q. codeletion	Codel vs. noncodel	3.15E−14	3.73E−29	1.94E−19	8.01E−5
Primary therapy outcome	CR vs. PD	7.01E−2	1.70E−4	6.00E−6	1.00
	CR vs. SD	1.00	1.00	0.0659	0.908
	CR vs. PR	0.540	1.00	1.00	1.000
	PD vs. SD	0.120	9.61E−3	6.02E−2	0.627
	PD vs. PR	1.00	0.0796	1.11E−4	0.336
	SD vs. PR	0.750	1.00	6.01E−6	1.00
Gender	Female vs. Male	0.038	0.106	0.606	0.355
Age	<=60 vs. >60	4.92E−20	4.81E−13	1.83E−14	0.149
OS	Alive vs. Dead	2.44E−26	4.9E−25	2.43E−31	0.110
DFS	Alive vs. Dead	0.030	1.90E−3	9.30E−3	0.420

Abbreviations: CR, complete response; PR, partial response; SD, stable disease; PD, progressive disease.

### 3.3 Coexpressed genes of *SOCS1/2/3/4* and gene and protein networks

**FIGURE 5:**
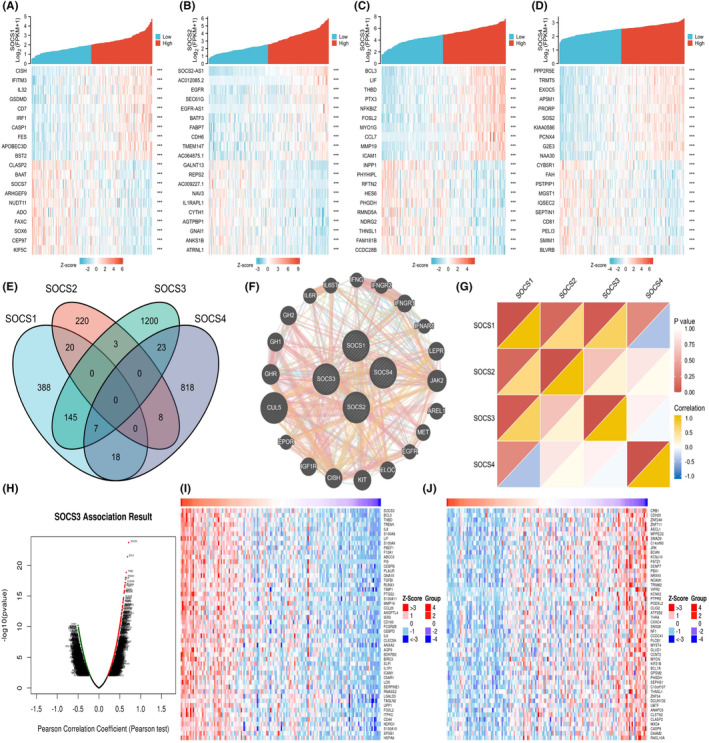
(A–D) The heat map showed the upregulated and downregulated top 10 differentially expressed genes (DEGs). Positive and negative DEGs of *SOCS1* (A), *SOCS2* (B), *SOCS3* (C), *SOCS4* (D) according to heat map and Venn diagram. (E) Intersection representing DEGs of *SOCS1/2/3/4*. |*r*| > 0.4 and *p* < 0.001. (F) Gene network associated with *SOCS1/2/3/4* and interactions between proteins encoded by genes of *SOCS1/2/3/4* using GeneMANIA. (G) Correlation analysis for *SOCS1/2/3/4*. (H) The total significantly associated genes with *SOCS3* distinguished by Pearson test in the TCGA‐GBM cohort. (I,J) Top 50 positively and negatively associated with *SOCS3* in TCGA‐GBM were shown, respectively, via heat maps. Red, positively linked genes; blue, negatively linked genes.

Analysis of TCGA database revealed 224 positively correlated and 230 negatively correlated genes for *SOCS1*; 380 positively correlated and 66 negatively correlated genes for *SOCS2*; 876 positively correlated and 470 negatively correlated genes for *SOCS3*; and 3988 positively correlated and 189 negatively correlated genes for *SOCS4*. The top 10 coexpressed genes that were positively and negatively correlated with *SOCS1/2/3/4* are shown in a heat map (Figure 5A–D). Furthermore, a Venn diagram showed seven intersections of *SOCS1/3/4* coexpressed genes (Figure 5E). The PPI network, which was generated by the GeneMANIA website, revealed that 20 potential target genes interacted with *SOCS1/2/3/4* (Figure 5F). Next, correlation analysis of *SOCS1/2/3/4* was performed using the cBioPortal database,^28^ and the results revealed that *SOCS1* and *SOCS3* were negatively correlated with SOCS4, while other genes were positively correlated (Figure 5G). As *SOCS3* has the greatest prognostic value for GBM, we explore the coexpressed genes of *SOCS3* in GBM, the Linked Omics database was used to analyse the *mRNA* sequencing analysis of 153 cases of patients with GBM in TCGA. The results showed that there were more differentially expressed genes positively correlated with *SOCS3* than negatively correlated (Figure 5H–J). showed 50 genes that were positively or negatively correlated, respectively, with *SOCS3*. Notably, *SOCS3* was most strongly correlated with *BCL3*, *THBD* and *TREM1*, with Pearson correlation coefficients of 0.6854, 0.6526 and 0.6415, respectively. For example, as a proto‐oncogene, *BCL3* is closely associated with the NF‐κB signalling pathway and is a member of the IκB family. Therefore, *SOCS3* may be involved in the progression of GBM through the NF‐κB signalling pathway. PPI network analysis showed that there were 20 genes associated with *SOCS1/2/3/4*, and 10 representatively hub genes were selected via correlation analysis, including *CUL5*, *IL6R*, *IL6ST*, *IFNGR1*, *IFNGR2*, *IFNAR1*, *AREL1*, *MET*, *EGFR* and *CISH* (Table S1). GEPIA^29^ analysis of these 10 genes showed increased expression of *IL6ST*, *IFNGR1*, *IFNGR2*, *IFNAR1*, *EGFR* and *CISH* in GBM (*p* < 0.05) (Figure S1). In addition, *CUL5*, *IL6R*, *IFNGR1*, *IFNGR2*, *IFNAR1*, *AREL1*, *MET*, *EGFR* and *CISH* were associated with the OS (months) of patients with GBM (Figure S2A). GBM patients with high transcriptional levels of *IL6R*, *IFNGR1*, *IFNGR2*, *IFNAR1*, *MET*, *EGFR* and *CISH* were significantly associated with short OS, while GBM patients with high transcriptional levels of *CUL5* and *AREL1* were related to longer OS. On the contrary, *IFNGR1*, *IFNGR2*, *IFNAR1*, *AREL1*, *MET* and *CISH* were associated with the disease‐free progression (DFS) (months) of patients with GBM (Figure S2B). GBM patients with high transcriptional levels of *IL6ST*, *IFNGR1*, *IFNGR2*, *IFNAR1*, *MET* and *CISH* were significantly associated with short DFS, while GBM patients with high transcriptional levels of *AREL1* were related to longer DFS.

### 3.4 GSEA analysis

**FIGURE 6:**
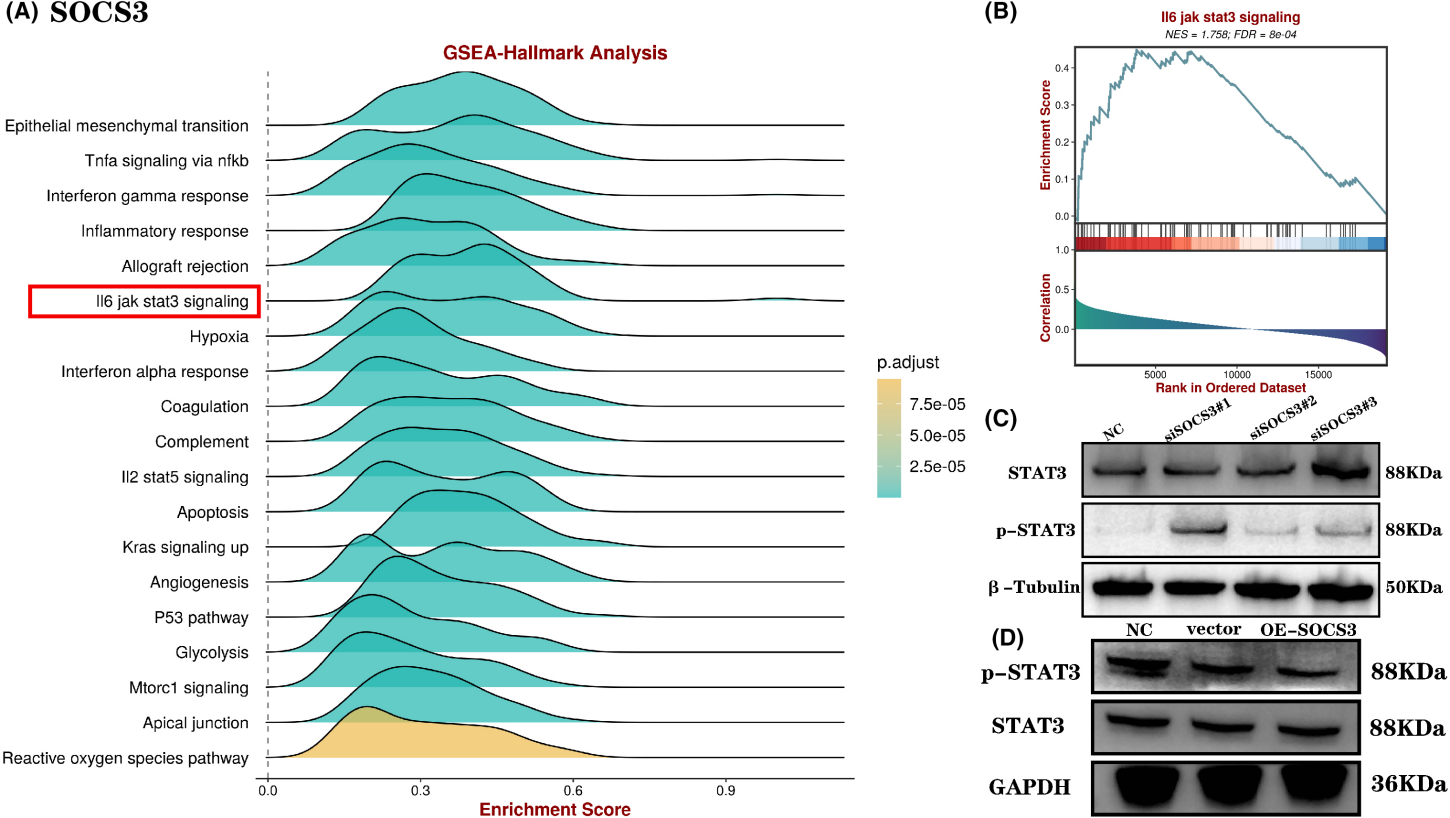
(A) GSEA analysis was employed to explore cancer hallmarks that enriched high and low *SOCS3* expression. (B) GSEA showed that IL‐6/JAK/STAT3 signalling pathway was differentially enriched in *SOCS3*. (C,D) Western blot revealed the relationship between *SOCS3* and JAK/STAT3 signalling pathway.

Since *SOCS3* has the greatest prognostic value in GBM, we also further performed enrichment analysis for *SOCS3*, GSEA (gene set enrichment analysis) was performed to identify the functional enrichment of high *SOCS3* expression and low *SOCS3* expression (Figure 6A). Notably, we found that *SOCS3* were associated with *IL‐6/JAK/STAT3* signalling pathway in GBM. Our further enrichment analysis of *SOCS3* found that high‐risk group was significantly associated with *IL‐6/JAK/STAT3* (Figure 6B). In order to confirm this idea, we used siRNA and plasmid to knock down and overexpress *SOCS3* in LN229 cells, respectively. Cells were collected to extract proteins, and *STAT3* and *p‐STAT3* antibodies were incubated with Western blot to observe the protein expression in different treatment groups. The results showed that as a suppressor of cytokine signalling 3, knockdown of *SOCS3* significantly increased the expression of *p‐STAT3* (Figure 6C), while overexpression of *SOCS3* decreased the expression of *p‐STAT3* (Figure 6D). The results showed that *SOCS3* may regulate the progression of GBM via *JAK/STAT3* signalling pathway.

### 3.5 Correlation of *SOCS1/2/3/4* expression with immune characteristics

**FIGURE 7:**
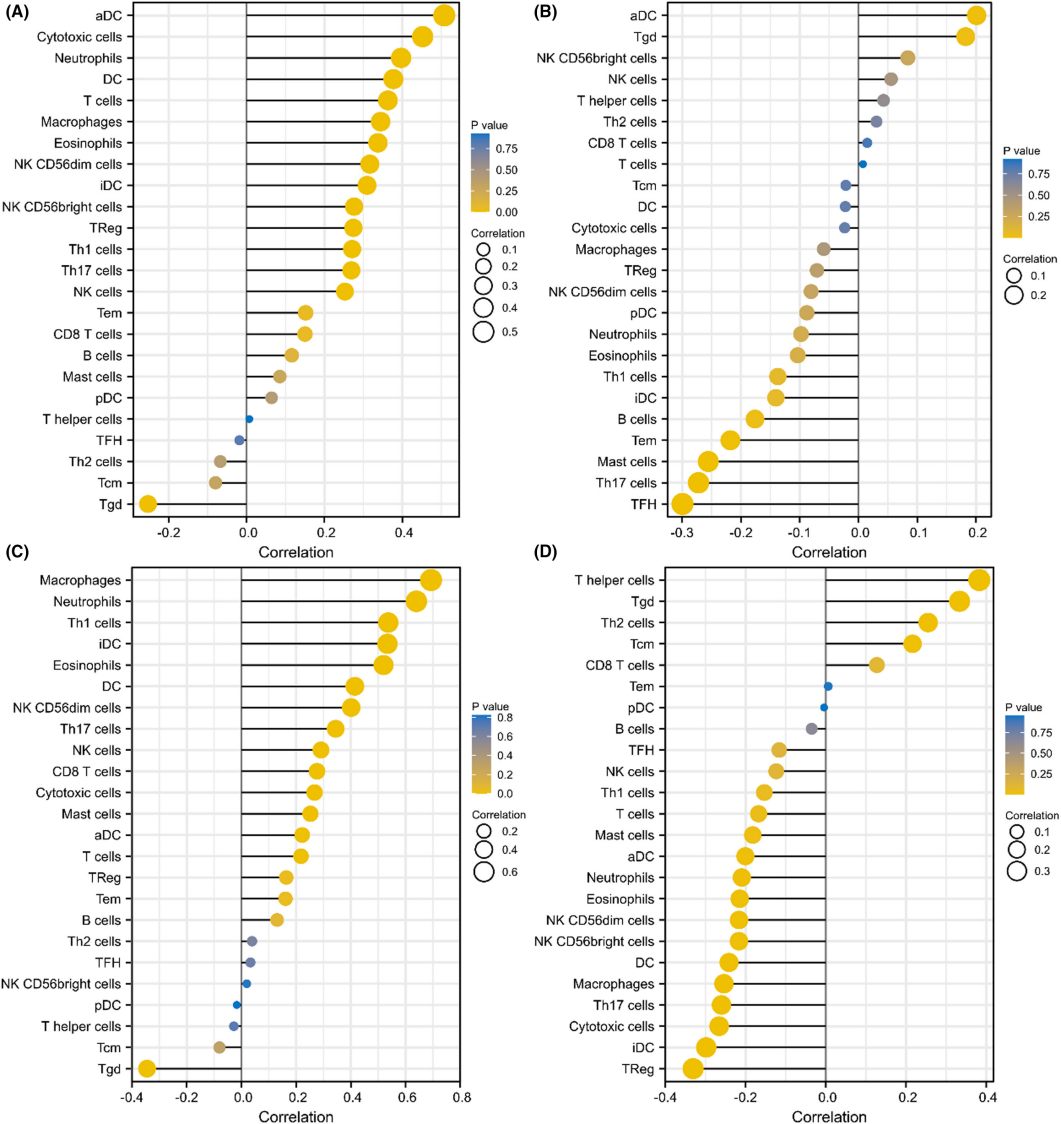
Correlation of *SOCS1/2/3/4* expression with immune infiltration in GBM using XianTao academic and TIMER2.0. (A–D) Correlation between the expression of *SOCS1/2/3/4* and multiples tumour‐infiltrating lymphocytes (TILs) in GBM.

Previous studies have shown that tumour infiltrating lymphocytes (TILs) are independent predictors in tumours.^30^ The present study analysed the association between *SOCS1/2/3/4* gene level and tumour‐infiltrating immune cells by using the XianTao academic (https://www.xiantao.love) and TIMER2.0 databases, and found that *SOCS1/2/3/4* affects tumour‐infiltrating immune cells in GBM (Figure 7). As shown in Figure 7, *SOCS1/2/3/4* are associated with a wide variety of immune cells. For instance, *SOCS1* expression was positively correlated with immune cells, including a DC, neutrophils and cytotoxic cells, but negatively correlated with Tgd cells. *SOCS2* expression was negatively correlated with several immune cell types, including Mast cells, Th17 cells and B cells, and was positively correlated with a DC cells. *SOCS3* expression was positively correlated with macrophages and neutrophils, but negatively correlated with Tgd cells. Furthermore, *SOCS4* expression was negatively correlated with Treg, iDC, macrophage and Th17 cells, while it was positively correlated with T helper cells and Tgd cells. Next, the association between *SOCS1/2/3/4* expression and abundance of 28 TILs was analysed via the TISIDB database, and Figures [Link], [Link] show the association between *SOCS1/2/3/4* expression and TILs in different types of tumours. In GBM, *SOCS1/2/3/4* expression was significantly associated with multiple TILs.

**FIGURE 8:**
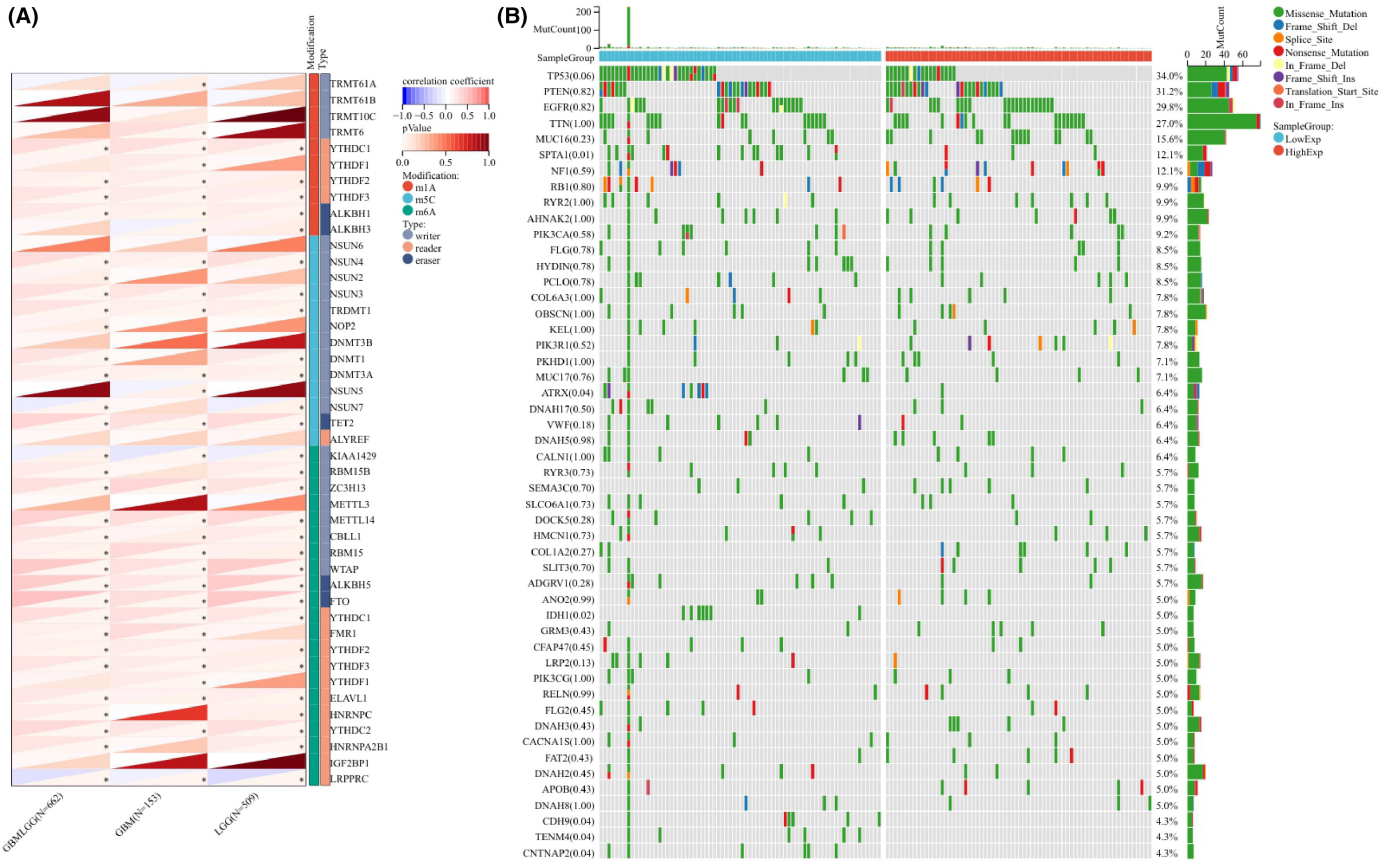
Analysis of epigenetic modification and mutation landscape. (A) Analysis of SOCS3 and epigenetic modification. (B) Analysis of SOCS3 and mutation landscape.

### 3.6 Analysis of SOCS3 and immunoregulation‐associated genes and immune cell infiltration

**FIGURE 9:**
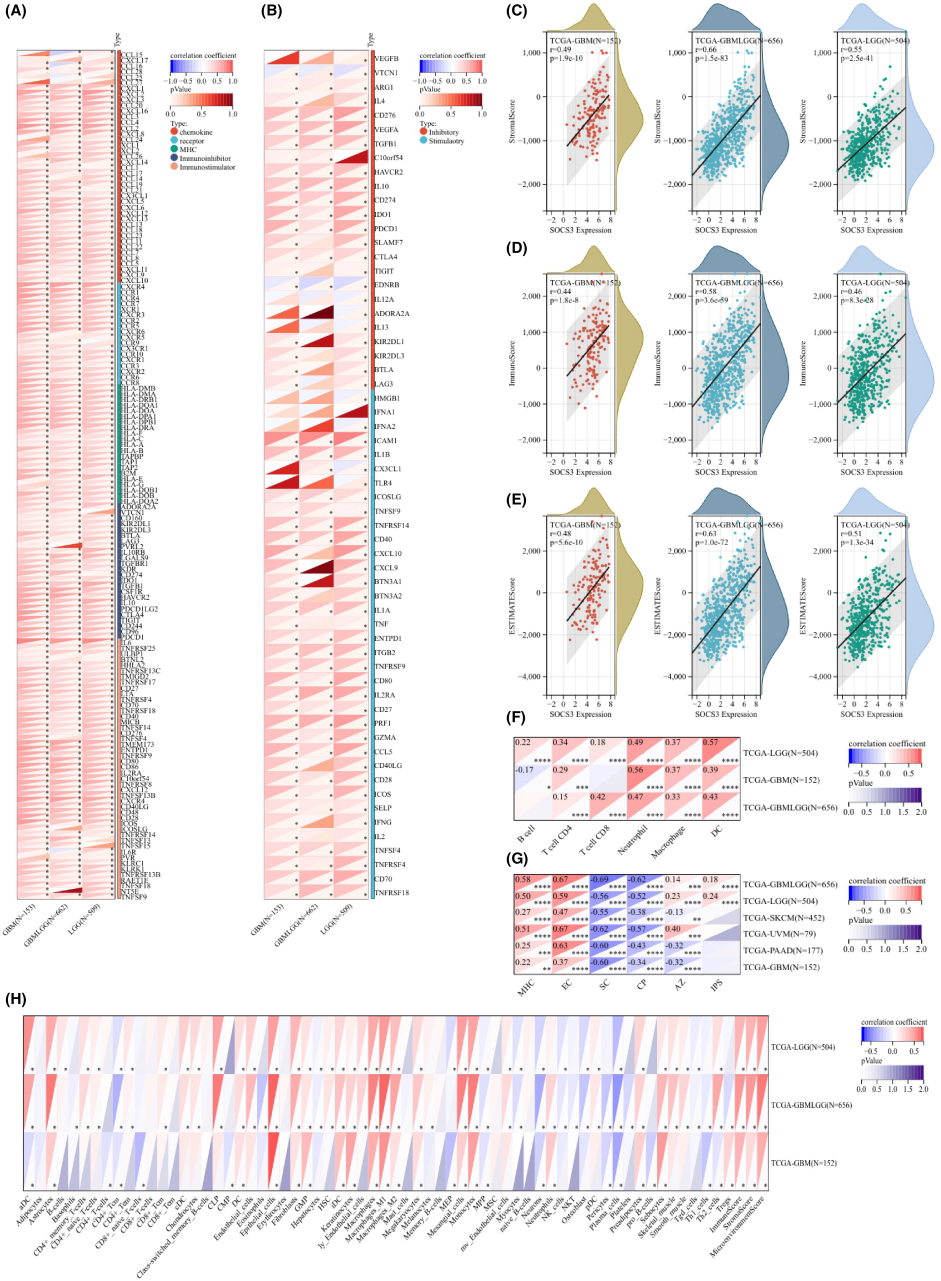
The relationship between *SOCS3* and immunoregulation‐associated genes and immune cell infiltration. (A,B) Analysis of immunomodulatory gene and immune checkpoint gene. (C,D) Analysis of immune cell infiltration. (E–H) Analysis of immune cell via Timer and xCELL.

The above analysis revealed that *SOCS1/2/3/4* expression was significantly correlated with multiple TILs. In order to further analyse the relationship between *SOCS3* and immune regulatory genes, immune checkpoint genes and immune cell infiltration, we searched Sangerbox3.0 website based on TCGA and GTEx databases. Since immune checkpoint genes provide important targets for tumour immunotherapy, we specifically analysed the correlation between immunomodulatory genes and immune checkpoint genes and glioma. We found that *SOCS3* was positively correlated with most of the related genes in glioma, including immune activation genes, immune checkpoint genes, chemokine genes and chemokine receptor genes (Figure 9A,B). We downloaded the agreed standardized pan‐cancer data set from the UCSC database (http://xenabrowser.net/),^31^ extracted the expression data of *SOCS3* in each sample, further screened the samples, applied log_2_ (x + 0.001) transformation to each expression value, extracted the glioma expression profile and mapped the expression profile to GeneSymbol. Stromal, immune and ESTIMATE scores of glioma patients were calculated based on gene expression using the R package ESTIMATE (version 1.0.12, http://bioinformatics.mdanderson.org/public‐software/estimate/). Finally, the immune infiltration scores of glioma samples were obtained, and the corr.tes function of R package psych (version 2.1.6) was used to calculate Pearson's correlation coefficient between *SOCS3* and immune infiltration scores in glioma. Finally, *SOCS3* was found to be significantly positively correlated with immune infiltration in gliomas, including GBM (Figure 9C–E). Based on the Timer and xCELL databases, we further analysed the correlation between *SOCS3* and immune cells in GBM, and the results showed that *SOCS3* was positively correlated with macrophages, neutrophils and DC cells, but negatively correlated with B cells (Figure 9F–H), which was consistent with the results in Figure 7C. However, Figure 9G reveals that *SOCS3* in GBM is positively correlated with major histocompatibility complex (MHC) and effector cells (EC), while negatively correlated with suppressor cells (SC) and others. Collectively, these results reveal the potential role of *SOCS3* and immune‐related genes in GBM and provide a reference for further research by subsequent researchers.

### 3.7 SOCS3 expression and epigenetic modification and mutation landscape

To explore the role of *SOCS3* in epigenetic modification, *SOCS3* was analysed at the epigenetic genome level. We used the cBioPortal database to explore the categories and frequencies of *SOCS3* gene alterations in different types of cancer. The results showed that *SOCS3* gene amplification and mutation are the main types of genetic alterations, especially hepatocellular carcinoma, breast cancer and melanoma. *SOCS3* gene amplification was the main type of genetic alteration in glioma (Figure S7A). The main types of *SOCS3* mutations are missense and truncation mutation (Figure S7B). Copy number values were significantly associated with diploid, gain and shallow deletion alterations of *SOCS3* (Figure S7C). RNA methylation is a common epigenetic modification and includes N1‐methyladenosine (m1A), cytosine‐5‐methylation (m5C) and N6‐methyladenosine (m6A). We analysed their correlation with *SOCS3* expression levels. As shown in Figure 8A, there was a significant positive correlation between *SOCS3* and 44 RNA modifications (m1A,^32^ m5C^32^ and m6A^32^) in the majority of human gliomas with statistical significance. *SOCS3* mutation frequencies were analysed in high and low expression groups in Figure 8B, and 50 genes with the highest mutation frequencies were identified. Mutation frequency reaches more than 10% of genes including *TP53* (34.0%), *PTEN* (31.2%), *EGFR* (29.8%), *TTN* (27.0%), *MUC16* (15.6%), *SPTA1* (12.1%) and *NF1* (12.1%), The most common type of mutation is missense mutation. It can be seen that the mutation frequency of *TP53*, *PTEN*, *EGFR*, *TTN*, *MUC16*, *SPTA1* and *NF1* genes is significantly increased, suggesting that *SOCS3* may promote the occurrence and development of GBM.

### 3.8 Verification of *SOCS1/2/3/4* *mRNA* expression in glioma tissue samples

The *mRNA* expression of *SOCS1/2/3/4* in 174 cases of GBM and noncancer tissues was analysed by TCGA database, and the association between *SOCS1/2/3/4 mRNA* expression and clinicopathological features is shown in Table 3. It was found that age and IDH status were the common influencing factors of *SOCS1/2/3/4 mRNA* levels. In addition, the *SOCS1 mRNA* levels were associated with OS and DFS, while the *SOCS2 mRNA* levels were associated with gender, race, Karnofsky performance score, OS and DFS. The *mRNA* level of *SOCS3* was associated with Karnofsky performance score, OS and DFS, while the *mRNA* level of *SOCS4* was markedly associated with Karnofsky performance score.

**TABLE 3:** Association between *SOCS1/2/3/4* and clinicopathological parameters in patients with GBM.

Clinicopathological parameters		N	SOCS1 expression (2 − ΔCq)		SOCS2 expression (2 − ΔCq)		*SOCS3* expression (2 − ΔCq)		SOCS4 expression (2 − ΔCq)	
			Mean ± SD	*p* value	Mean ± SD	*p* value	Mean ± SD	*p* value	Mean ± SD	*p* value
Gender	Male	109	3.439 ± 0.981	0.221	2.839 ± 1.192	0.007	4.735 ± 1.371	0.172	2.487 ± 0.374	0.285
	Female	59	3.521 ± 1.079	0.366	2.776 ± 1.412	0.003	4.946 ± 1.316	0.152	2.496 ± 0.366	0.457
Age (years)	≤60	87	1.939 ± 0.828	0.077	2.777 ± 1.239	0.019	6.393 ± 1.561	0.034	4.044 ± 0.490	0.010
	>60	81	2.197 ± 0.842	0.001	2.859 ± 1.308	0.001	6.567 ± 1.303	0.243	3.995 ± 0.473	0.861
Race	Asian	5	1.847 ± 0.868	0.207	3.016 ± 1.885	0.711	3.922 ± 1.324	0.901	2.867 ± 0.137	0.266
	Black or African American	11	1.982 ± 0.661	0.711	2.163 ± 1.280	0.044	4.480 ± 1.418	0.315	2.323 ± 0.501	0.498
	White	150	2.081 ± 0.858	0.002	2.876 ± 1.239	0.000	4.855 ± 1.351	0.013	2.487 ± 0.359	0.471
Karnofsky performance score	<80	36	2.148 ± 0.755	0.063	2.891 ± 1.264	0.094	4.782 ± 1.209	0.885	2.568 ± 0.345	0.408
	≥80	92	2.009 ± 0.880	0.106	2.906 ± 1.288	0.016	4.893 ± 1.415	0.024	2.493 ± 0.359	0.009
IDH	WT	149	2.141 ± 0.826	0.000	2.899 ± 1.275	0.000	4.942 ± 1.283	0.115	2.468 ± 0.368	0.488
	Mut	12	1.231 ± 0.748	0.022	2.029 ± 0.972	0.159	3.206 ± 1.561	0.023	2.672 ± 0.284	0.023
OS	Alive	32	1.804 ± 0.790	0.314	2.832 ± 1.401	0.018	4.777 ± 1.067	0.642	4.217 ± 0.453	0.178
	Dead	136	2.124 ± 0.845	0.001	2.813 ± 1.242	0.002	4.816 ± 1.414	0.025	3.974 ± 0.477	0.231
DFS	Alive	34	1.798 ± 0.788	0.129	2.798 ± 1.366	0.010	4.821 ± 1.048	0.635	2.629 ± 0.382	0.050
	Dead	121	2.090 ± 0.822	0.002	2.790 ± 1.229	0.004	4.813 ± 1.441	0.019	2.456 ± 0.362	0.169

### 3.9 Analysis of *SOCS1/2/3/4* protein expression level by IHC staining

**FIGURE 10:**
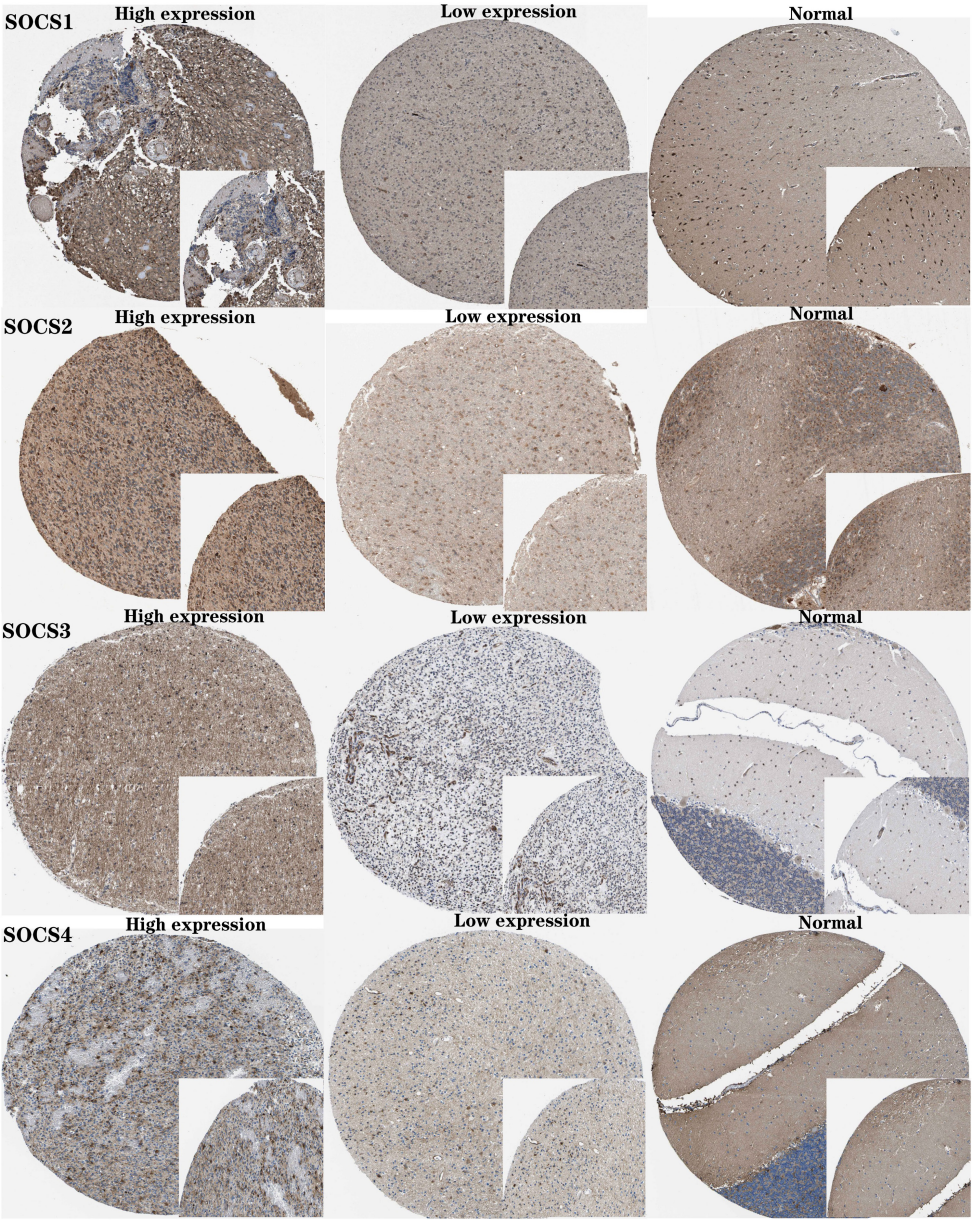
The immunohistochemistry (IHC) results from the Human Protein Atlas (HPA) was used to explore the protein level of *SOCS1/2/3/4* genes in normal and glioma tissues. The figure revealed the alteration of *SOCS1/2/3/4* protein expression levels in glioma (low grade and high grade) and normal brain tissue.

The HPA database for immunohistochemical staining was employed to explore the protein levels of *SOCS1/2/3/4* in glioma samples (low grade and high grade) and normal brain tissue. The results revealed that *SOCS1/2/3/4* protein expression in high‐grade glioma tissues were higher than those in LGG and normal tissues. The expression level of *SOCS1/2/3/4* in normal tissues was the lowest (Figure 10). These findings suggested that upregulation of *SOCS1/2/3/4* may predict advanced malignancies such as GBM.

### 3.10 Experimental verification of the fact that *SOCS3* is highly expressed in GBM cell lines and patients' tissues

**FIGURE 11:**
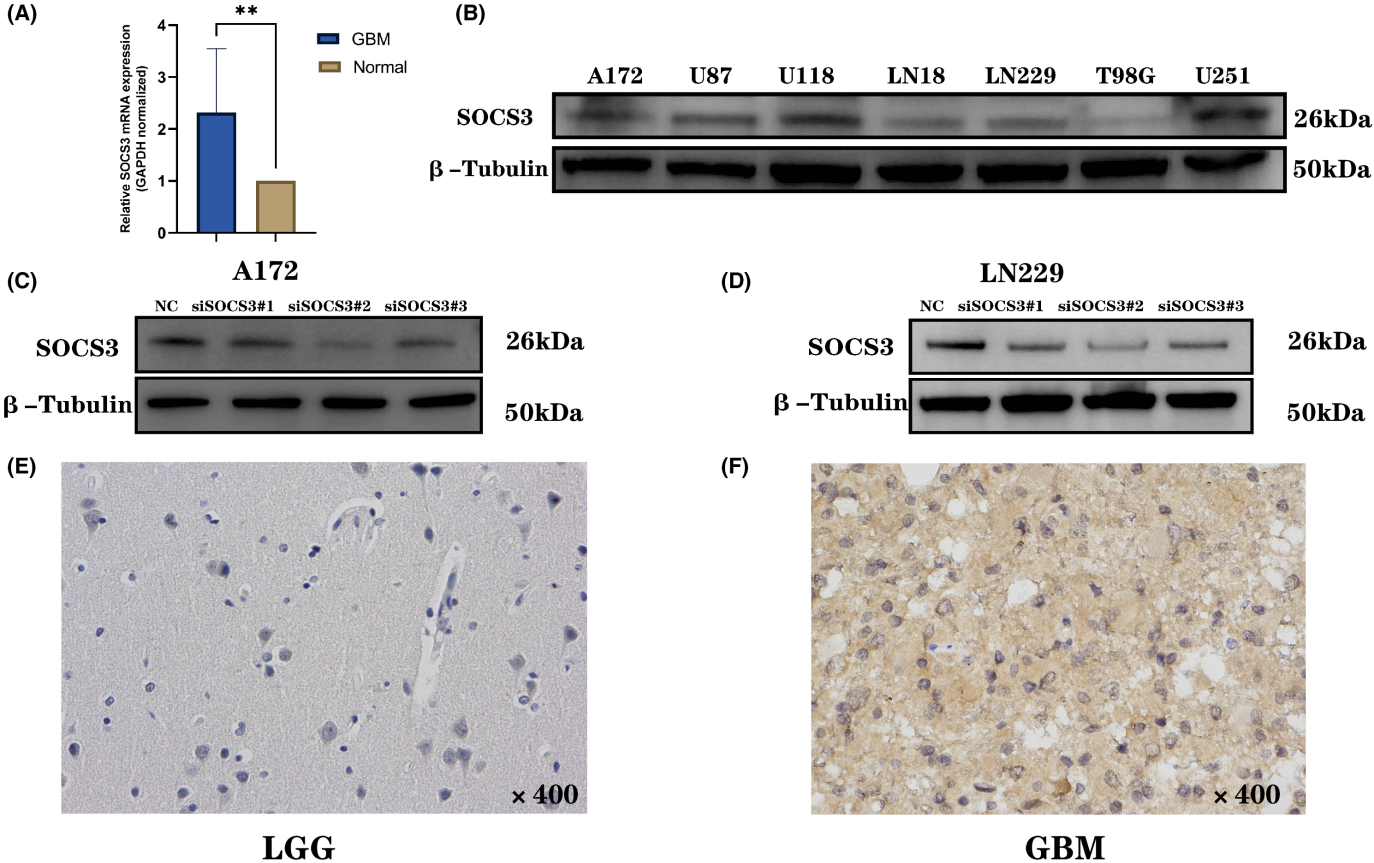
*SOCS3 mRNA* and protein expression in GBM cell line and human tissue. (A) qRT‐PCR was employed to explore the *mRNA* expression of *SOCS3* in GBM and normal tissues, (B) Western blotting was employed to evaluate the protein expression of *SOCS3* in GBM cell lines, (C,D) A172 and LN229 cells were transfected with siRNA, and the knockdown efficiency was evaluated by Western blotting. The results of each interference experiment were repeated more than three times. (E,F) IHC was used to explore *SOCS3* expression in LGG and GBM human tissue specimens. **p < 0.01.

To further verify the aforementioned bioinformatics analysis results, a series of experiments were carried out. RNA was extracted from 14 tissue specimens for the PT‐qPCR analysis, and it was found that patients with GBM had higher *SOCS3 mRNA* expression than those found in normal tissues (Figure 11A). Western blotting was performed on seven human GBM cell lines, and it was found that A172, U87, U118, LN229 and U251 cells had higher *SOCS3* expression levels (Figure 11B). Therefore, A172 and LN229 cells were transfected with siRNAs and the knockdown efficiency was statistically significant, particularly for LN229 (Figure 11C,D). IHC staining revealed that *SOCS3* was highly expressed in nine of 11 tissues derived from patients with GBM, while *SOCS3* expression was low in all 12 patients with LGG (Figure 11E,F). In conclusion, the present study experimentally confirmed that *SOCS3* exhibited high *mRNA* and protein levels in GBM cell lines and tissues derived from patients with GBM.

### 3.11 Knockdown of *SOCS3* attenuates GBM cells proliferation, migration and invasiveness

**FIGURE 12:**
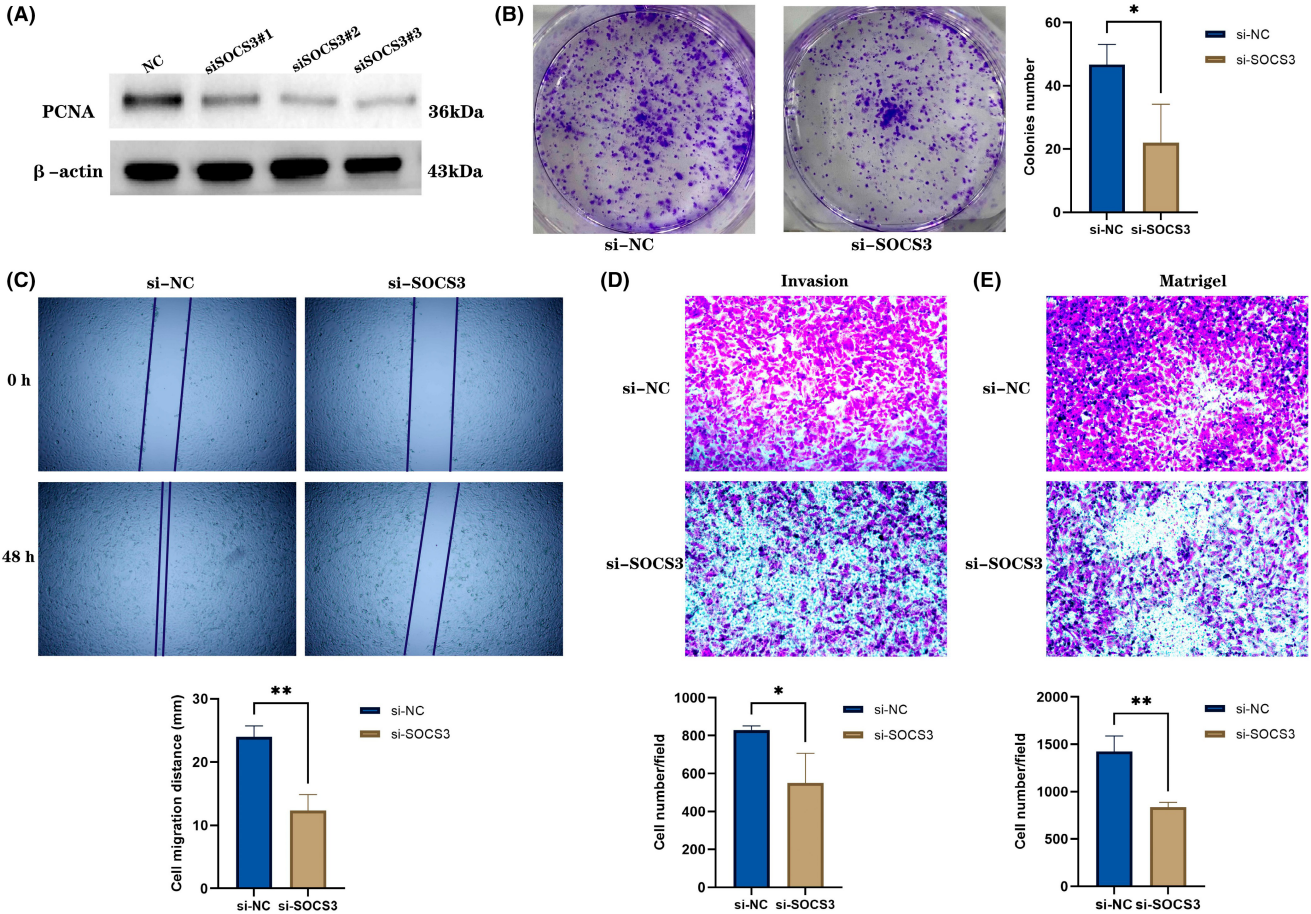
(A,B) Western blotting and colony formation assay were performed to explore the influence of *SOCS3* on GBM cell proliferation. (C–E) Wound healing assay and Transwell assays were employed to explore the influence of *SOCS3* on GBM cell migration and invasion. **p < 0.01, *p < 0.05.

To identify the functional role of *SOCS3* in GBM, LN229 cells with higher transfection efficiency were selected for further experiments. As aforementioned above, siRNA was used for transfection and Western blotting was performed to verify the knockdown efficiency. Numerous studies have revealed that *SOCS3* expression is closely associated with the proliferation of tumour cells.^33^, ^34^ The current study used *SOCS3* as a marker of cell proliferation for knockdown experiments, and detected PCNA expression by Western blotting, which revealed that the expression level of PCNA was reduced (Figure 12A). In addition, transfected cells were plated into 6‐well plates for colony formation assay, and the cell proliferation of groups subjected to different treatments was observed 10 days later. It was found that the cell proliferation of *SOCS3* knockdown group was significantly reduced (Figure 12B). Wound healing and Transwell assays revealed that the migration and invasion abilities of LN229 cells with *SOCS3* knockdown were markedly reduced (Figure 12C–E). In conclusion, inhibition of *SOCS3* expression could reduce the proliferation, migration and invasion of GBM cell lines.

## 4 DISCUSSION

GBM is a malignant brain tumour with a high mortality rate and a high degree of malignancy. In recent years, the number of studies on molecular biomarkers and their effects on tumour treatment, survival and prognosis has increased. Davis et al revealed the molecules and pathways associated with the pathogenesis of GBM through gene mapping, which may help to develop new treatment methods for GBM.^35^

Using TCGA and GEO data sets as well as various databases, we found that *SOCS1/2/3/4* were high expressed in brain and CNS cancer and lymphoma, while low expressed in bladder cancer, gastric cancer, liver cancer, lung cancer, ovarian cancer and prostate cancer. To further explore the expression of SOCS protein in brain tumours, TCGA‐GTEx database was used to analyse the *mRNA* expression levels of *SOCS1/2/3/4*, respectively, the results demonstrated that *SOCS1/2/3/4* expression level was higher in GBM than in normal samples. High *mRNA* expression was associated with WHO grade, histological type, 1p/19q co‐deletion, therapeutic effect and prognosis. The higher the WHO grade of glioma, the higher the expression of *SOCS1/2/3/4* and the worse the survival and prognosis of patients, which was statistically significant in *SOCS1/2/3*. TCGA database was used to explore *SOCS1/2/3/4* coexpressed genes, construct PPI networks and analyse the effects of coexpressed genes on the OS and DFS of patients with GBM. The present study further performed GSEA enrichment analyses of *SOCS1/2/3/4* coexpression genes and found that *SOCS1/2/3/4* coexpression was mainly involved in JAK/STAT signalling pathway.

The survival and prognosis of glioma patients are closely associated with a variety of clinicopathological parameters. Our study found that the expression of *SOCS1/2/3* increased with the increase of WHO grade of glioma, especially the abnormal expression of *SOCS3* in high‐grade glioma. The expression of *SOCS1/2/3* in GBM was significantly higher in gliomas of different histological classifications than in other histological types, while the expression of *SOCS4* was not significantly different in gliomas of different histological classifications. In addition, *SOCS1/2/3/4* expression is closely associated with 1p/19q co‐deletion, and *SOCS1/3/4* expression is associated with IDH status, age and overall survival of glioma patients. In conclusion, *SOCS1/2/3/4* is associated with a variety of clinicopathological parameters in glioma, especially *SOCS3*, which provides guidance for us to improve the prognosis of glioma patients.

Genetic and epigenetic alterations are known to affect gene expression and may also be associated with adverse clinical outcomes.^22^ We mainly analysed the types and frequencies of *SOCS3* gene alterations in cancers, and found that gene mutation and amplification were the main types of alterations, and *SOCS3* mutations were mainly missense mutations and truncation mutations. In glioma, *SOCS3* is positively correlated with 44 RNA modifications (m1A, m5C and m6A), and the most common type of mutation is missense mutation, and the mutation frequency of *TP53*, *PTEN*, *EGFR*, *TTN*, *MUC16*, *SPTA1* and *NF1* genes significantly increased. Overall, *SOCS3* may contribute to the occurrence and development of GBM.

The tumour immune microenvironment is associated with the therapeutic and prognostic response of tumours.^36^, ^37^ The current study revealed that *SOCS1/2/3/4* expression was associated with immune cell infiltration in GBM. Specifically, *SOCS1* was positively correlated with CD4^+^ T cells, neutrophils, myeloid dendritic cells and other immune cells, but negatively correlated with tumour purity. *SOCS2* expression was negatively correlated with CD4^+^ T cells, neutrophils and B cells, and positively correlated with tumour purity. *SOCS3* expression was positively correlated with macrophages and neutrophils, but negatively correlated with B cells and tumour purity. *SOCS4* expression was negatively correlated with macrophages and NK cells, but positively correlated with CD4+ T cells and tumour purity. The present study found that *SOCS1* and *SOCS3* expression levels were associated with GBM tumour‐promoting immune cells, while *SOCS2* and *SOCS4* were not. These results provide knowledge about *SOCS3* expression, tumour immunotherapy and prognosis in GBM.

*SOCS* proteins are an intracellular cytokine‐inducible proteins that inhibit the JAK/STAT signalling pathway.^38^ The *SOCS* protein family consists of eight members, namely *SOCS1–SOCS7* and *CIS*. Each protein member contains an SH2 domain and a specific *SOCS* box. Notably, *SOCS1* and *SOCS3* possess a kinase inhibitory region (KIR), which is the main functional domain.^39^ The present study revealed that the *mRNA* and protein expressions levels of *SOCS3* in GBM cell lines and patients with GBM were higher than those in normal tissues and LGG. Moreover, high SOCS3 expression was associated with poor overall survival, disease‐specific survival and progression‐free survival in GBM patients. These results suggest that the *SOCS* family, particularly *SOCS3*, may be a potential diagnostic and prognostic tool that promotes carcinogenesis in patients with GBM.

At present, *SOCS* proteins play important roles in regulating tumour progression in a variety of cancers. Zhou et al. found that exosome miR‐155 secreted by melanoma cells promotes the expression of receptor fibroblast proangiogenic factors via the *SOCS1*/JAK2/STAT3 signalling pathway, thereby inducing the preangiogenic switch of CAFs. Therefore, exosome *miR‐155* may be a potential target to control the angiogenesis of melanoma.^40^ As a *METTL3*‐mediated m6A modification target, m6A modification of *SOCS2 mRNA* disappears with *METTL3* knockdown, thus promoting the increase of *SOCS2 mRNA* expression and ultimately inhibiting the progression of hepatocellular carcinoma.^41^ Studies have found that IL‐23 causes the 3'‐UTR binding of *miR‐25* and *SOCS4* by upregulation of miR‐25 expression, resulting in the inhibition of *SOCS4*, and ultimately promoting the migration and invasion of thyroid cancer cells.^42^ Zhang et al. found that *SOCS3* promoted cell proliferation and invasion in non‐small cell lung cancer by regulating the expression of Pyk2.^43^ It has also been found that *EYA2* is downregulated in hepatocellular carcinoma (HCC), and *EYA2* can regulate *SOCS3* expression in combination with *DACH1* transcription, thereby inhibiting HCC progression via *SOCS3*‐mediated blockade of the JAK/STAT signalling pathway.^44^ Current studies have found that the high expression of *SOCS3* can promote the proliferation of GBM cells.^34^ Therefore, we want to further study the other functions of *SOCS3*, including migration and invasion, and explore the potential mechanisms of *SOCS3*'s possible regulatory role by bioinformatics analysis, so as to prepare for further exploration of *SOCS3* mechanisms in GBM in the future.

The current study found that GBM with high *SOCS1/2/3/4* expression had a poor prognosis. Our group previously found a potential correlation between *SOCS3* expression and GBM.^45^ The current study verified the presence of high *SOCS3* expression in a variety of cell lines through cell experiments, and the RT‐qPCR and IHC analyses of human tissue specimens demonstrated the high expression of *SOCS3* in GBM. To further explore the potential role of *SOCS3* as a marker for poor prognosis of GBM, *SOCS3* was knocked down by siRNA, and it was found that cell lines with reduced *SOCS3* expression had lower proliferation levels, and reduced migration and invasion abilities. In conclusion, *SOCS3* may be a potential prognostic marker for patients with GBM.

In conclusion, *SOCS3* was identified as a predictor of survival in patients with GBM by analysing RNA‐based gene expression profiles from TCGA data sets and online databases. Moreover, the present findings verified high *SOCS3* expression in GBM cell lines and human tissues through in vitro experiments, and demonstrated that GBM cell lines with high *SOCS3* expression exhibited higher proliferation, migration and invasion abilities.

## AUTHOR CONTRIBUTIONS

**Lirui Dai:** Conceptualization (equal); data curation (lead); formal analysis (lead); investigation (lead); methodology (lead); resources (lead); validation (lead); visualization (lead); writing – original draft (lead); writing – review and editing (lead). **Yongjie Han:** Formal analysis (supporting); validation (supporting); writing – review and editing (supporting). **Zhuo Yang:** Data curation (supporting); formal analysis (supporting); methodology (supporting); resources (supporting). **Yuling Zeng:** Methodology (equal); validation (equal). **Wulong Liang:** Conceptualization (supporting); funding acquisition (equal); project administration (supporting); resources (supporting); supervision (supporting); validation (supporting). **Zimin Shi:** Validation (equal); writing – review and editing (equal). **Yiran Tao:** Validation (supporting); writing – review and editing (equal). **Xianyin Liang:** Validation (supporting); writing – review and editing (supporting). **Wanqing Liu:** Resources (supporting); validation (supporting). **Shaolong Zhou:** Funding acquisition (equal); resources (supporting). **Zhe Xing:** Methodology (supporting); validation (supporting). **Weihua Hu:** Project administration (supporting); supervision (supporting). **Xinjun Wang:** Conceptualization (lead); funding acquisition (lead); supervision (lead).

## FUNDING INFORMATION

The present study was supported by grants from the National Natural Science Foundation of China (grant nos. 81972361 and 81874068) and Medical Science and Technology Project of Henan Province (grant nos. LHGJ20210487 and 222102310039).

## CONFLICT OF INTEREST STATEMENT

The authors declare no potential competing interests.

## CONSENT

The patients/participants provided their written informed consent to participate in this study.

## Supporting information


Figure S1

Figure S2

Figure S3

Figure S4

Figure S5

Figure S6

Figure S7

Table S1

## Data Availability

All data included in this study are available by contacting the corresponding authors.
