# Supplementary figures and images for: Identification and validation of SOCS1/2/3/4 as potential prognostic biomarkers and correlate with immune infiltration in glioblastoma
Source: J Cell Mol Med. 2023 Jun 14;27(15):2194–214. doi: 10.1111/jcmm.17807 (PMC10399539; doi:10.1111/jcmm.17807)

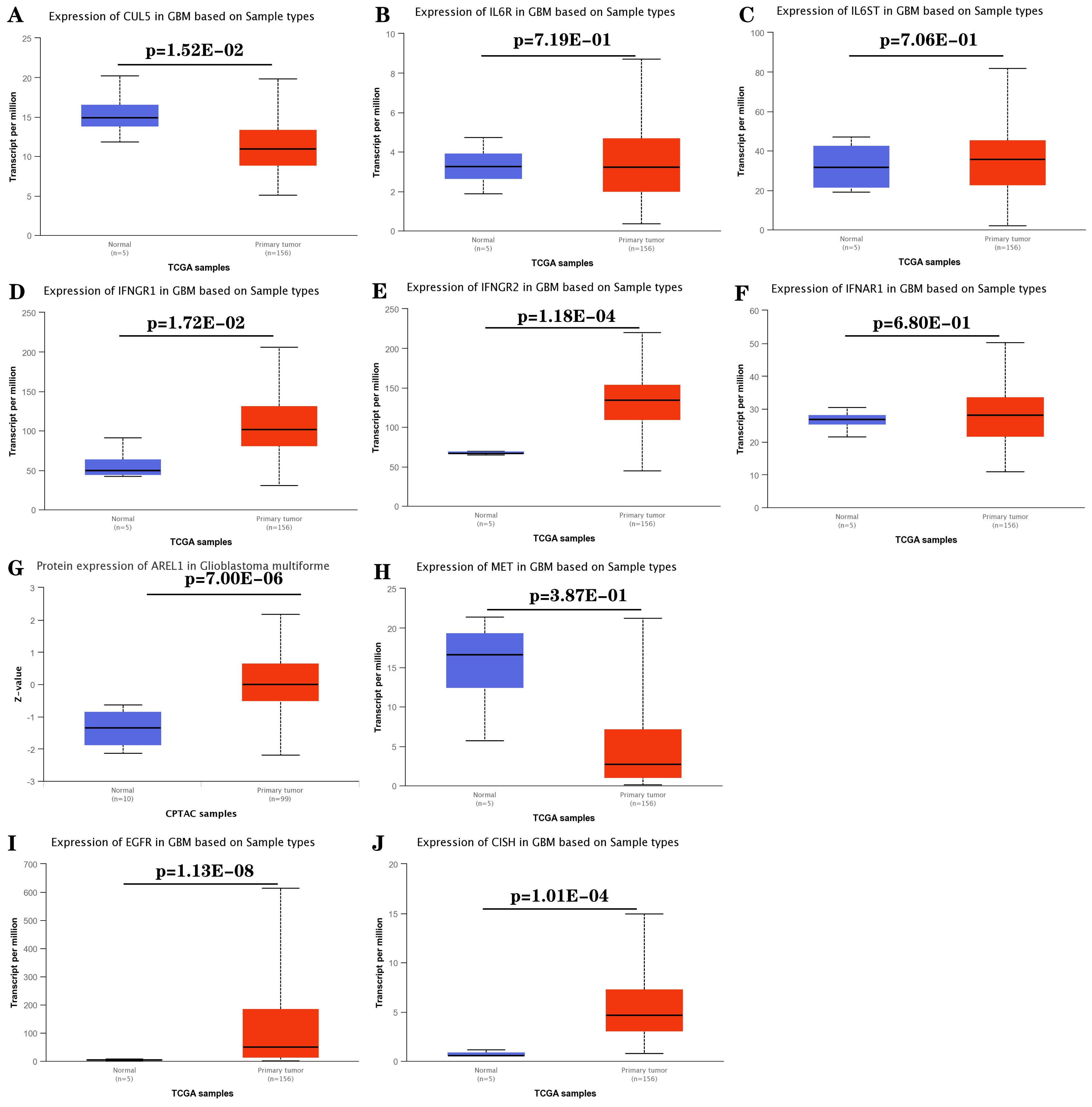

Supplement: Supplementary file 1 — Figure S1 [file JCMM-27-2194-s007.jpg]

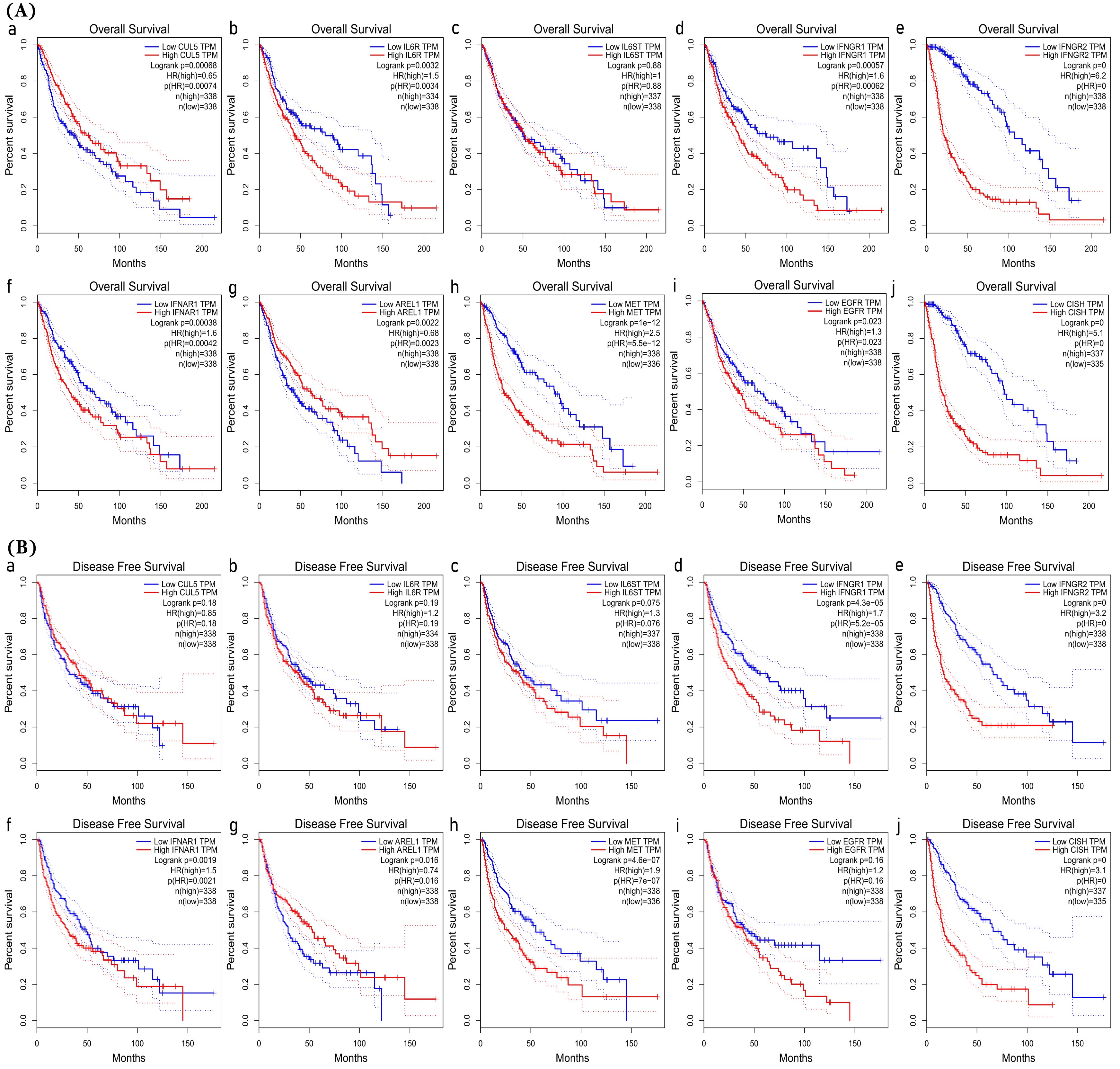

Supplement: Supplementary file 2 — Figure S2 [file JCMM-27-2194-s006.jpg]

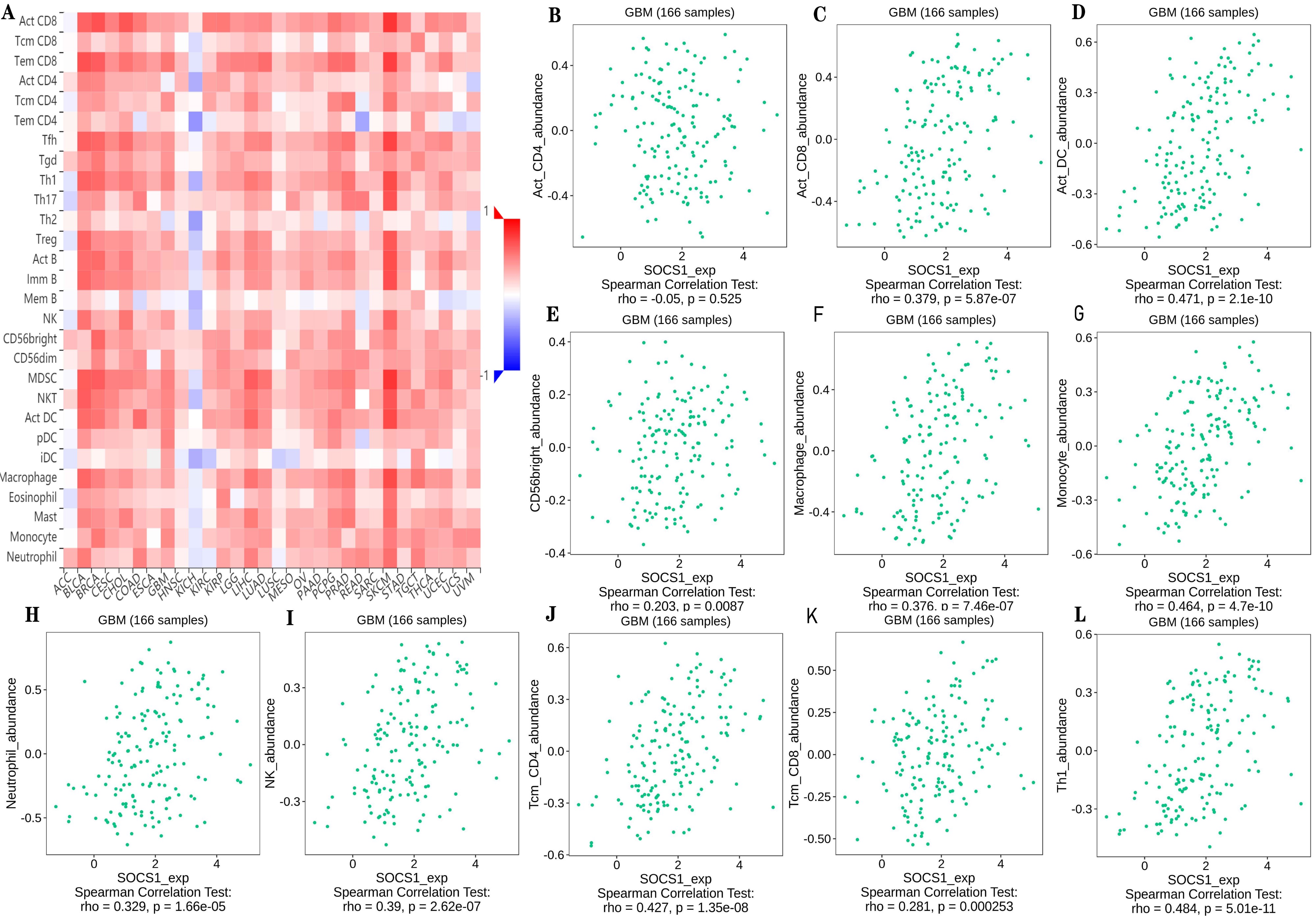

Supplement: Supplementary file 3 — Figure S3 [file JCMM-27-2194-s008.jpg]

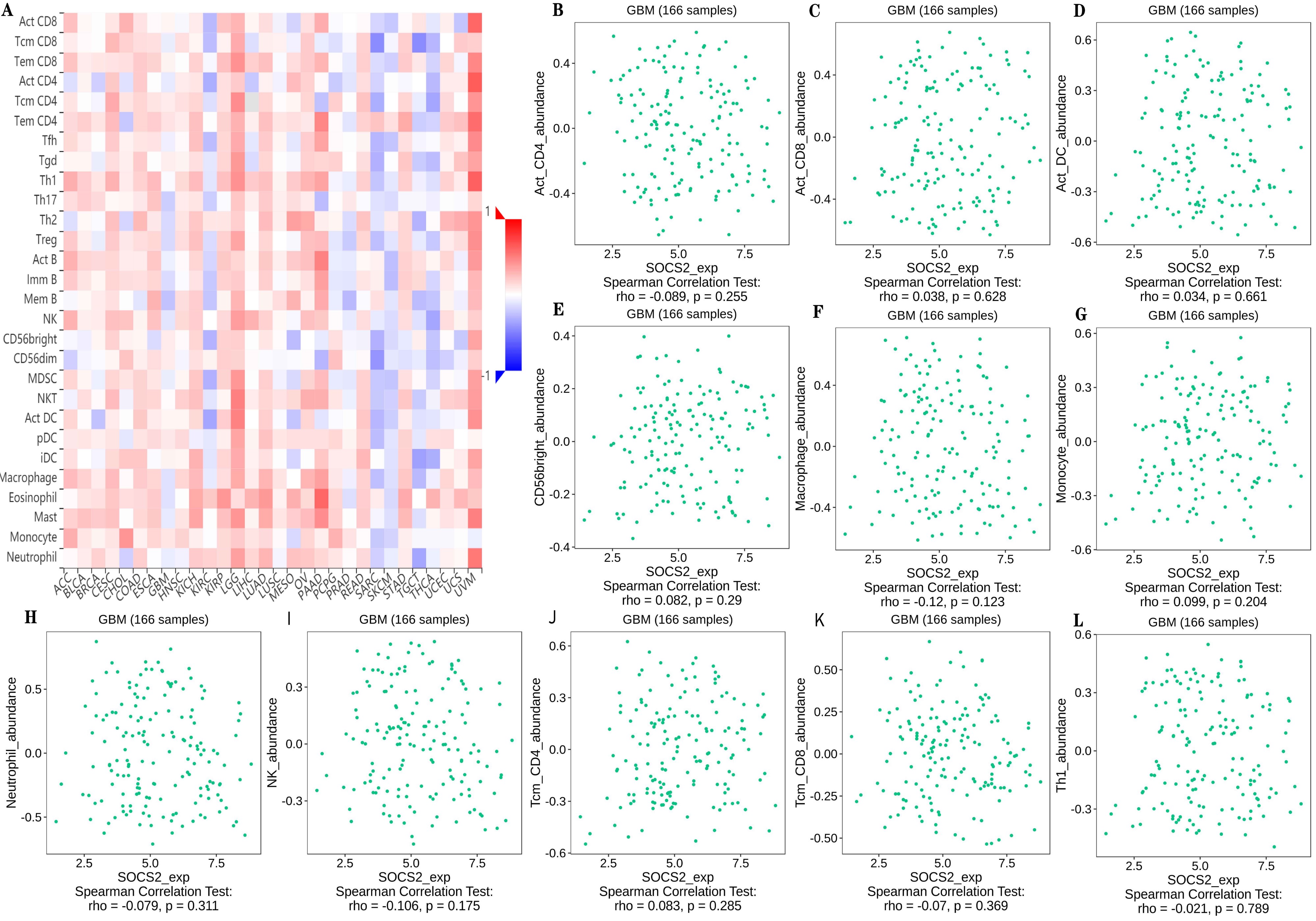

Supplement: Supplementary file 4 — Figure S4 [file JCMM-27-2194-s005.jpg]

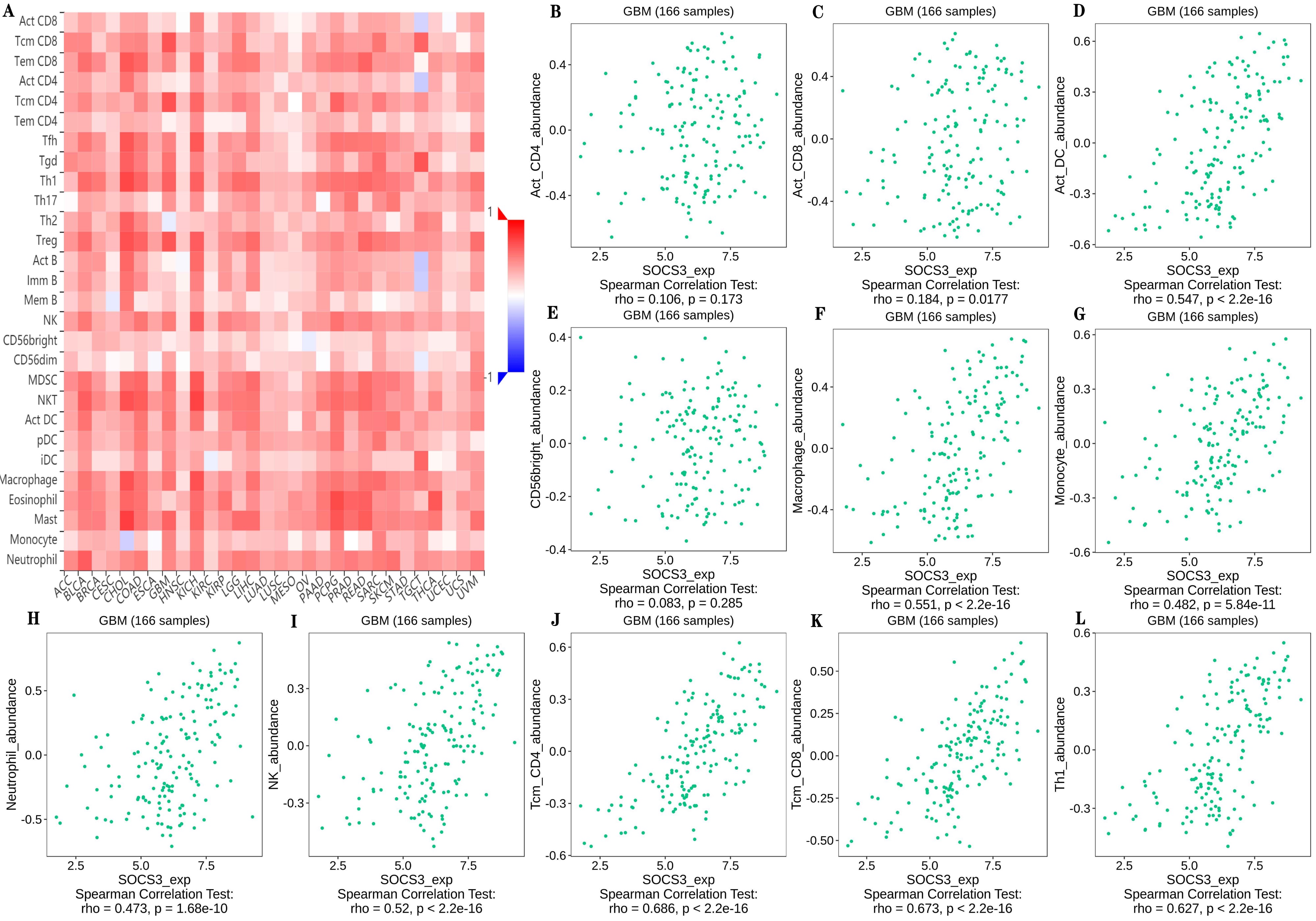

Supplement: Supplementary file 5 — Figure S5 [file JCMM-27-2194-s002.jpg]

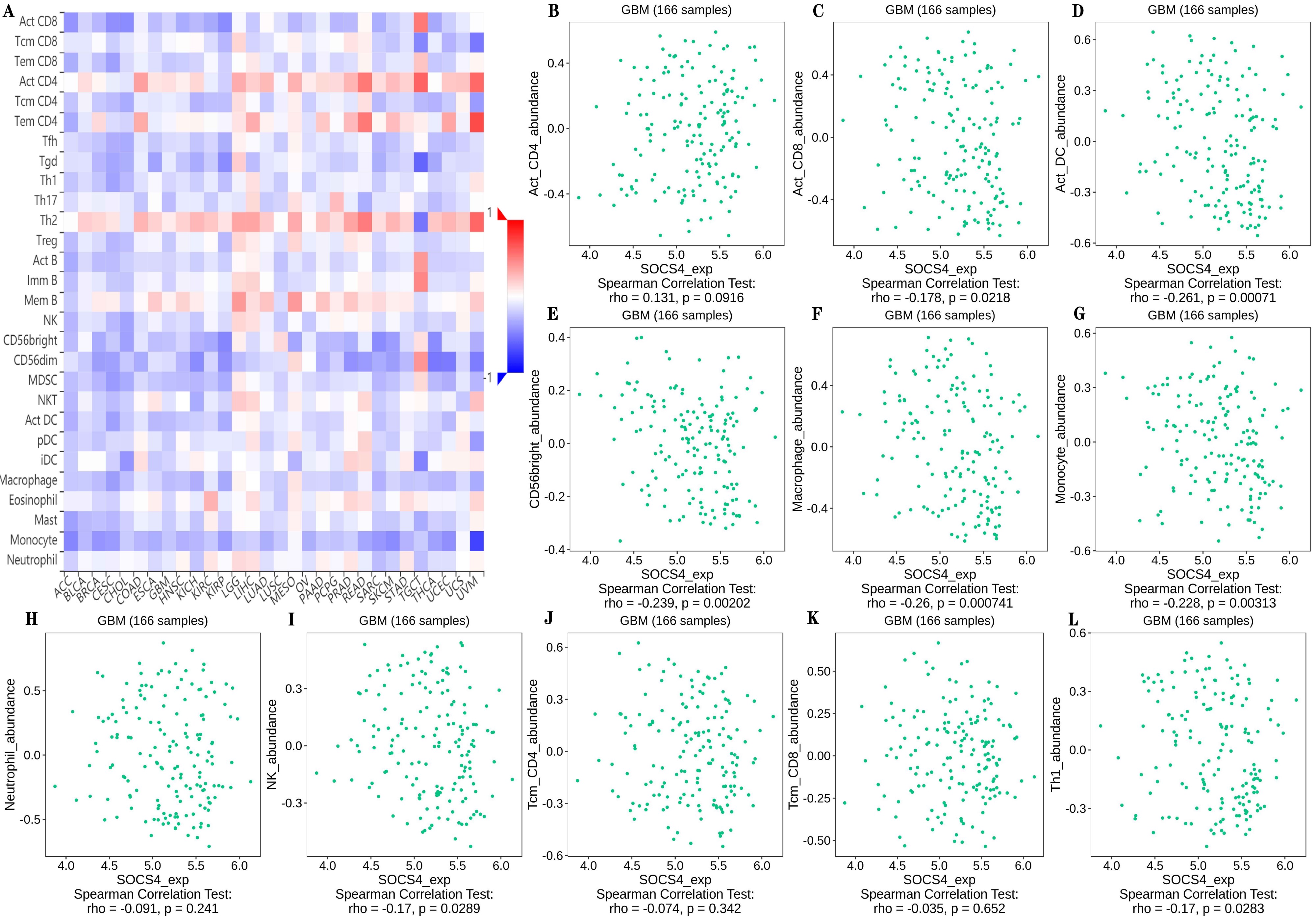

Supplement: Supplementary file 6 — Figure S6 [file JCMM-27-2194-s003.jpg]

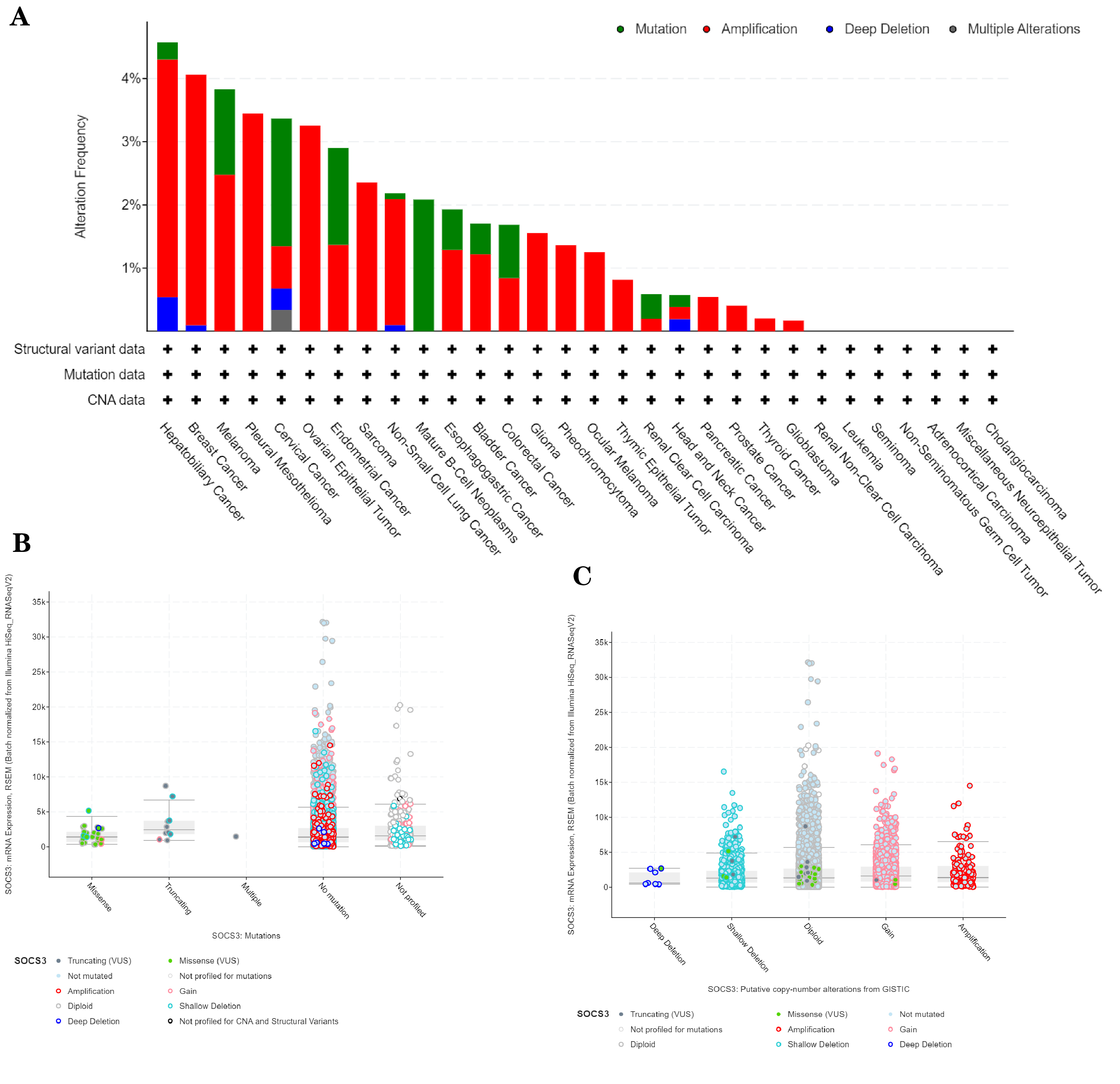

Supplement: Supplementary file 7 — Figure S7 [file JCMM-27-2194-s001.jpg]
