# Supplementary material for: Identification and validation of SOCS1/2/3/4 as potential prognostic biomarkers and correlate with immune infiltration in glioblastoma
Source: J Cell Mol Med. 2023 Jun 14;27(15):2194–214. doi: 10.1111/jcmm.17807 (PMC10399539; doi:10.1111/jcmm.17807)
Supplement: Supplementary file 8 — Table S1 [file JCMM-27-2194-s004.docx]

**TABLE SⅠ.** The correlations between the representative 10 Hub genes with SOCS1/2/3/4 in GBM patients

| Gene symbol | Gene description | SOCS1(r) | SOCS2(r) | SOCS3(r) | SOCS4(r) |
| --- | --- | --- | --- | --- | --- |
| CUL5 | Cullin 5 | -0.260 | 0.033 | -0.160 | 0.340 |
| IL6R | Interleukin 6 Receptor | 0.250 | -0.190 | 0.430 | -0.130 |
| IL6ST | Interleukin 6 Cytokine Family Signal Transducer | 0.130 | 0.200 | 0.270 | 0.140 |
| IFNGR1 | Interferon Gamma Receptor 1 | 0.120 | 0.083 | 0.064 | -0.059 |
| IFNGR2 | Interferon Gamma Receptor 2 | 0.430 | 0.250 | 0.540 | -0.026 |
| IFNAR1 | Interferon Alpha and Beta Receptor Subunit 1 | 0.099 | 0.034 | 0.480 | 0.200 |
| AREL2 | - | -0.220 | -0.052 | -0.014 | 0.570 |
| MET | MET proto-oncogene, receptor tyrosine kinase | 0.098 | -0.320 | 0.480 | -0.250 |
| EGFR | Epidermal Growth Factor Receptor | 0.200 | 0.570 | -0.220 | 0.260 |
| CISH | Cytokine Inducible SH2 Containing Protein | 0.640 | 0.390 | 0.410 | -0.061 |
